# Genome-wide identification, characterization and expression pattern analysis of HAK/KUP/KT potassium transporter gene family in potato

**DOI:** 10.3389/fpls.2024.1487794

**Published:** 2025-01-16

**Authors:** Kun Liu, Yonglin Qin, Lan Wu, Rong Yi, Xiaohua Shi, Jing Yu, Xiaohong Shi, Wenzhuo Dong, Liguo Jia, Mingshou Fan

**Affiliations:** College of Agronomy, Inner Mongolia Agricultural University, Hohhot, Inner Mongolia, China

**Keywords:** potato, HAK/KUP/KT, potassium ion transport, gene expression, potassium response

## Abstract

The HAK/KUP/KT (High-affinity K^+^ transporters/K^+^ uptake permeases/K^+^ transporters) is the largest and most dominant potassium transporter family in plants, playing a crucial role in various biological processes. However, our understanding of HAK/KUP/KT gene family in potato (*Solanum tuberosum* L.) remains limited and unclear. In this study, 24 *HAK/KUP/KT* genes (*StHAKs*) were identified through a genome-wide analysis and were found to be unevenly distributed across ten chromosomes. Based on phylogenetic analysis, these *StHAK* gene family members were classified into four distinct clusters. All StHAK protein sequences contained the conserved motifs and domains. Promoter cis-acting elements analysis revealed that most *StHAK* gene family members in potatoes were associated with responses to light and hormones such as abscisic acid or methyl jasmonate, however, many motifs responsive to hormones and stress conditions have not been clearly studied or reported in plants. Synteny analysis suggested that 33, 19, 8, 1 *StHAK* genes were orthologous to those in soybean, cassava, Arabidopsis and rice, respectively. The previously published RNA-seq results, transcriptomic data and qRT-PCR experiments indicated that the expression profiles of these *StHAK* genes were tissue-specific and were influenced by multiple factors, including biotic and abiotic stress, hormone, potassium fertilizer. To provide a clear and convenient view of *StHAK* gene expression across different tissues in potato, we generated a cartoon heatmap to vividly illustrate the tissue-specific expression of *StHAK* genes, which is unprecedented in the gene family analysis of potato. At last, we identified genes such as *StHAK8*, *StHAK14*, and *StHAK22* with high expression in potato tubers using qRT-PCR, suggesting their potential involvement in tuber growth and development. This can contribute to a deeper understanding of the mechanism of potassium absorption and transportation in potatoes. It has laid a solid theoretical foundation for the genetic regulation of potassium nutritional efficiency in potatoes and the breeding of potato varieties with high potassium efficiency.

## 1 Introduction

As one of the most abundant and essential mineral nutrient element in plant, potassium regulates numerous physiological and metabolic procedures, and most importantly, it determines the yield and quality of crop production (Sardans and Peñuelas, 2021). Due to its high mobility in plants, potassium is involved in regulating critical metabolic pathways and contributing to synthesis and transport of proteins and sugars (Fan et al., 2019). Additionally, potassium is engaged in stabilizing balance between anion and cation and in regulating cellular osmotic potential, strengthening the water content of protoplasm and enhancing the drought resistance of plant (Torabian et al., 2021). After wheat (*Triticum aestivum*), rice (*Oryza sativa*) and maize (*Zea mays*), potato (*Solanum tuberosum* L.) is the fourth most important crop in world and has a great demand for potassium, which is the predominant element in ash, accounting for 50-70% of the total ash content (Fan et al., 2019).To produce one ton of tubers, potatoes need to absorb 4.0-6.0 kg of K_2_O, 1.0-1.5 kg of P_2_O_5_, and 3.0-4.0 kg of N from the soil (Chen et al., 2024; Kong et al., 2024; Shi et al., 2019). A sufficient supply of potassium strengthens stems, making them more robust and resilient, thereby enhancing resistance to collapse, freezing, and diseases in potatoes. Additionally, it promotes tuber bulking and significantly improves potato yield (Shi et al., 2019). Some quality index, such as protein, starch and vitamins in tubers, can also be improved with adequate potassium supply, and the tuber hollow rate in potatoes is significantly reduced (Naumann et al., 2020). With insufficient potassium, potato stolons are significantly shortened, and root development and nutrient absorption are noticeably decreased (Fan et al., 2019).

In plants, K^+^ is primarily absorbed by the roots through dual-affinity mechanisms, which include high-affinity and low-affinity systems for potassium assimilation. When the external potassium concentration is below 0.2 mM, a high-affinity transport system (HATS) is activated, facilitating the movement of K^+^ from the extracellular to the intracellular environment via potassium transporters. Conversely, when the external potassium concentration exceeds 0.3 mM, a low-affinity transport system (LATS) via potassium channels is initiated (Li et al., 2018). For optimal plant function, the K^+^ concentration in various cellular compartments, including the nucleus, cytosol, chloroplast stroma, and mitochondrial matrix, should be maintained between 100 and 150 mM (Britto and Kronzucker, 2008). The minimum vacuolar and tissue concentration of K^+^ present in cell are approximately 10 mM and 5 mg K (g dry matter (DM))^-1^, respectively (White, 2013). In many cultivated soils, potassium application is insufficient to maintain healthy growth and development of crop, with K^+^ concentration in soil typically varying between 0.01 and 1 mM, representing only 0.1%~0.2% of the total soil K (White, 2013). Consequently, the acquisition of K^+^ from the surrounding soil by plant roots is primarily mediated through HATS, while the translocation of K^+^ from root to shoot and its distribution from source to sink within the plants is predominantly mediated through both HATS and LATS (Wang et al., 2021).

In higher plants, K^+^ transport occurs primarily through potassium transporter and ion channel protein families, with K^+^ channel proteins encoded by three main families: Shaker-like K^+^ channels, tandem-pore K^+^ (TPK) channels, and K^+^ inward rectifiers (Kir). and K^+^ transporters classified into four families: *HAK/KUP/KT* (High-affinity K^+^ transporters/K^+^ uptake permeases/K^+^ transporters), *HKT* (High-affinity K^+^ transporters), *NHX* (Na^+^/H^+^ exchangers) and *CHX* (Cation/H^+^ exchangers) (Johnson et al., 2022). Among these transporter protein families, the KUP/HAK/KT family (abbreviated as HAK) is the largest and plays a crucial role in mediating intracellular K^+^ accumulation for maintaining plant growth and development (Wang et al., 2021). HAK functions as a K^+^/H^+^ symporter, facilitating K^+^ uptake in plants by linking high-affinity K^+^ transport with H^+^ gradients (Santa-Maria et al., 2018; Sze and Chanroj, 2018).The plant HAK/KUP/KT transporters were first identified from barley (*Hordeum vulgare*) (Santa-María et al., 1997). Since then, they have been discovered in a wide range of plant species, including rice, wheat, maize, sugarcane (*Saccharum spontaneum*), *Brassica napus*, grape (*Vitis vinifera*), tomato (*Lycopersicon esculentum*), among others (Zhou et al., 2020; Luo et al., 2021; Lei et al., 2022; Li et al., 2022; Run et al., 2022; Che et al., 2024; Wang et al., 2024);

Under K^+^-deficient conditions, *Arabidopsis* HAK/KUP/KT transporter *HAK5* is primarily expressed in the root and responsible for K^+^ absorbed from the rhizosphere soil (Jiménez-Estévez et al., 2024). Like *AtHAK1*, *AtKUP3*, *AtHAK5*, *HvHAK1*, *OsHAK1*, and *CaHAK1*, they also serve as highly efficient potassium transporters across various plant species (Feng et al., 2020). In addition to K^+^ uptake and transport, *HAK/KUP/KT* genes are also associated with stress response and tolerance. The expression levels of *CeqHAK11* and *CeqHAK6* shows a significant increase as the duration of salt treatment progressed, and overexpression of *CeqHAK6* or *CeqHAK11* in *Arabidopsis* shows higher germination, survival rates and longer root length than wild-type under salt stress (Wang et al., 2023). *OsHAK16* is induced by K^+^ deficiency or salt stress, and its knockout mutant exhibits significantly increased salt sensitivity (Feng et al., 2019). *ZmHAK4* can promote Na^+^ exclusion from shoots and improve salt tolerance, contributing to the development of salt-resistance maize varieties (Zhang et al., 2019). All *KT/HAK/KUPs* in *Citrullus lanatus* and *Citrullus amarus* exhibit specific expression responses to drought stress (Cheng et al., 2024). RT-qPCR data shows that all six *MsHAK* genes are highly induced by salt and drought treatment, and all RT-qPCR for six *MsHAK* genes are positively correlated with transcriptome data (Li et al., 2022).

Besides their roles in K^+^ acquisition, translocation and stress tolerance, members of *HAK/KUP/KT* genes family are also involved in regulating root morphogenesis and development. In the presence of 10 μM K^+^, roots of F130S-1 and F130S-2 lines (Both strains are mutant lines of the *AtHAK5* gene) shows significantly larger roots than those of WT and *akt1* ones (63 and 66% larger with respect to akt1, respectively) (Jiménez-Estévez et al., 2024). The addition of K^+^ significantly stimulated adventitious root development, with roots beginning to emerge at 8 days and showing a marked increase in number at 16 days compared to the K^+^-free control. Notably, several *MdHAKs* demonstrated strong root-specific expression in stem cuttings of B9 apple rootstock, with their expression levels sharply upregulated during the initiation and emergence phases of adventitious root formation under K^+^ treatment (Tahir et al., 2023). *TINY ROOT HAIR 1* (*TRH1*), a HAK/KUP/KT transporter in *Arabidopsis* root cells, regulates intracellular ionic gradients and polar auxin transport, driving root epidermal differentiation and gravitropic responses (Templalexis et al., 2022). *AtKUP9*, highly expressed in roots and specifically expressed in quiescent center cells in root tips, controls primary root growth in *Arabidopsis*, and *kup9* displayed a short-root phenotype that resulted from reduced numbers of root cells under low-K^+^ conditions (Zhang et al., 2020). Although the crucial functions of *HAK* genes have been characterized in different plant species, the regulatory mechanisms of *HAK* in potato remain poorly understood, and a systematic study of the *HAK* gene family in potato is still lacking.

Currently, the HAK gene family has been reported in many plants (Guo et al., 2024). The *HAK* family genes are widely expressed in various tissues, including roots, stems, leaves, and tubers, enabling plants to efficiently absorb potassium in low-potassium environments, maintaining cellular ion balance and osmotic pressure, and regulating stress responses and metabolic processes, but the relationship between evolution and functional characterization of *HAK* family genes remains unclear (Jin et al., 2021; Johnson et al., 2022). Research in this field is particularly lacking in potato. In this study, a genome-wide analysis of *HAK* genes family in potato was conducted and 24 *HAK* gene family members were identified. The study also explored phylogenetic relationships, conserved motifs and domains, cis-regulatory elements, collinearity and syntenic relationships, as well as expression profiles across various tissues and potassium gradients to elucidate their potential biological functions in potato. Notably, our findings indicated that the *StHAK* genes exhibited distinct expression patterns across different tissues, and their transcription was induced by a range of biotic and abiotic stresses, including potassium availability. These studies will enhance our understanding of the functions of the *HAK/KUP/KT* gene family and have important implications for improving potassium utilization efficiency, as well as for advancing agricultural practices and production in potatoes.

## 2 Materials and methods

### 2.1 Identification of *StHAK* gene family in potato

In this study, the candidate *HAK* genes in potato were identified. The coding sequences (CDS), transcript sequences (cDNA), protein sequences and locus (genomic) sequences were download from Phytozome 13.0 (Goodstein et al., 2012) and Potato Genomic Resource (http://spuddb.uga.edu/). A profile hidden Markov model (pHMM) file for the ‘K_trans’ (Pfam: PF02705) domain was obtained from the Pfam protein family database (El-Gebali et al., 2018). The K_trans.hmm file was used to search against protein sequences from various plant genomic databases using the Hmmsearch program (HMMer package version 3.1b1). An *E*-value threshold of 1e^-10^ was set to identify plausible domains, and multiple sequence alignment was performed using Cluster W (V2.1). In consideration of the different predicted isoforms of genes loci, the primary isoforms were selected based on the annotation. If no annotation was available, the longest protein sequence was chosen for follow-up studies. To confirm the ‘K_trans’ domain in the predicted protein, sequences were upload to SMART (http://smart.emblheidelberg.de/), NCBI CDD (https://www.ncbi.nlm.nih.gov/cdd/) and HMMER (http://www.hmmer.org/) for online analysis (Finn et al., 2011; Letunic and Bork, 2018; Lu et al., 2020). Only genes with an intact ‘K_trans’ domain were retained for further analysis and research.

The isoelectric point (pI) and Molecular weight (Mw) of StHAKs were predicted by the ExPASy server (https://web.expasy.org/compute_pi/). Transmembrane domains (TMS) in the StHAK proteins were predicted by TMHHM Server v.2.0 (http://www.cbs.dtu.dk/services/TMHMM/). Conserved domains of the StHAK proteins were identified using the NCBI Conserved Domain Database (https://www.ncbi.nlm.nih.gov/Structure/bwrpsb/bwrpsb.cgi). Additionally, the subcellular localization of all StHAK proteins were predicted using WOLF PSORT (https://www.genscript.com/wolf-psort.html) (Horton et al., 2007).

### 2.2 Phylogenetic analysis of StHAK proteins

The full-length amino acid sequences of *HAK* genes identified from *S. tuberosum*, *A. thaliana*, *O. sativa* and *Z. mays* were aligned using ClusterW with default parameters (Larkin et al., 2007). A phylogenetic tree of the *HAK* gene family was then constructed using the neighbor-joining (NJ) method with 1000 bootstrap replicates in MEGA7.0 software. In addition, the phylogenetic relationships of HAKs were also analyzed using IQ-TREE with the maximum likelihood (ML) method. The resulting phylogenetic trees were decorated and visualized using the online tool iTOL (Letunic and Bork, 2007).

### 2.3 Gene structure and conserved motif analysis of *StHAKs*

The exon/intron organization of *StHAKs* was analyzed by comparing cDNA sequences with their corresponding genomic DNA sequences and visualized using the Gene Structure Display Server 2.0 (GSDS; http://gsds.cbi.pku.edu.cn/) (Hu et al., 2015). The conserved motifs in *StHAKs* were identified through Multiple Ems for Motif Elicitation (MEME) online program (http://meme-suite.org/ ) with the default parameters and the maximum number of conserved motifs was set to 10. The schematic paragraph of the phylogenetic tree, gene structure and conserved motifs were re-edited in PDF and assembled using TBtools software (Chen et al., 2023a).

### 2.4 Chromosomal distribution, gene duplication, collinearity and synteny analysis of *StHAKs*

The chromosomal distribution, gene duplication events, and collinearity analysis of the *StHAK* gene family were carried out using Advanced Cricos tool in TBtools (Chen et al., 2023a). The synteny relationships of *HAK* genes between potato and other plants were analyzed via Multiple Collinearity Scan toolkit (MCScanX) (http://chibba.pgml.uga.edu/mcscan2/) (Wang et al., 2012). The plant species included in the analysis were *Arabidopsis thaliana*, *Solanum lycopersicum*, *Glycine max*, *Manihot esculenta*, *Oryza sativa*, *Sorghum bicolor*, *Zea mays*, *Vitis vinifera*.

### 2.5 Analysis of cis-acting elements in *StHAK* genes promoter region

The 2000 bp of 5’ upstream *StHAK* CDS sequences were retrieved from the Spud DB online database (http://solanaceae.plantbiology.msu.edu/). These sequences were then submitted to the PlantCARE online program (http://bioinformatics.psb.ugent.be/webtools/plantcare/html/) (Lescot et al., 2002). We predicted cis-regulatory elements related to plant growth and development, phytohormone responsiveness, as well as stress response in the promoter regions of the *HAK* genes. Some cis-acting elements that were unrelated or lacked explicit annotations were excluded. The identified cis-regulatory elements were visualized using the Simple Biosequence Viewer program in TBtools.

### 2.6 Gene expression level analysis of *StHAK* genes based on microarray data

According to the Spud DB database (https://spuddb.uga.edu), expression profiles of *StHAK* genes were retrieved based on published RNA-seq data from 15 types of tissues, 3 types of abiotic stresses, 3 types of biotic elicitors and 4 types of hormonal treatments (Xu et al., 2011). The genomic locus information for *S. tuberosum* Group Phureja DM 1–3 v1.6 was used to analyze the expression levels of *HAK* genes. The gene expression values (Transcripts per million, TPM) from 219 potato Microarray libraries in the SRA were retrieved from the database. The tissues examined included sepals, leaves, roots, shoots, callus, tubers, stolons, petioles, petals, stamens, carpels, flowers, mature fruits, immature fruits, mesocarp & endocarp. Abiotic stresses contained NaCl (150 mM) for salinity treatment, mannitol (260 μM) for dehydration treatment, and 35°C for heat treatment. Biotic stresses included BABA (β-aminobutyric acid), BTH (benzothiadiazole), and pathogen, with leaves treated for 72 hours. The whole potato plants were incubated for 24 hours under four hormonal condition: 6-benzylaminopurine (BAP, 10 μM), indole-3-acetic acid (IAA, 10 μM), abscisic acid (ABA, 50 μM), and gibberellic acid (GA3, 50 μM). The expression matrix data for *StHAK* genes were used to generate a heatmap with the heatmap program in TBtools. For statistical convenience, each TPM value was log2-transformed (TPM+1).

### 2.7 Plant materials and different treatment

The potato research was conducted under field conditions between 2021 and 2022 in Siziwang County (41°46′N, 111°46′E) and County Chayouzhong (41°30′N, 112°64′E), Inner Mongolia Autonomous Region, China. The experimental sites are located in the Inner Mongolia plateau region, with an average altitude of approximately 1700 meters and an average annual temperature of 1.3 °C. The frost-free period in this region is about 100 d, and the annual evaporation exceeds 2000 mm (Cui et al., 2020). Precipitation at the trial sites during the growing season was approximately 250 mm in 2021 and 350 mm in 2022. The soil at the experimental sites is calcareous with a sandy texture. The basic physical and chemical properties of the 0~20 cm upper soil are detailed in **Supplementary Table 1**.

The experiments were preformed based on a randomized block design with three replicates per treatment. Each plot measured 72 m² (8×9 meters), with potato plants (cultivar Zhongjia-2) spaced 24–26 cm apart within rows, and rows separated by 90 cm. During the growing period, a drip irrigation system was extensively applied (Shi et al., 2019). Three potassium levels were tested in the field experiment: K0 (0 kg/hm^2^), K1 (300 kg/hm^2^), K2 (600 kg/hm^2^). In the controlled field experiment, nitrogen fertilizer was applied at 300 kg/hm^2^ as coated urea (N 46%), phosphorus fertilizer was applied at 180 kg/hm^2^ as triple superphosphate (P_2_O_5_ 50%), and potassium sulfate was used as the potassium source (K_2_O 50%). Nitrogen, phosphorus and potassium fertilizers were all broadcasted at sowing for each treatment. Potatoes were sown on May 12, 2021, and May 8, 2022, and harvested on September 3, 2021, and September 13, 2022, respectively.

Before the potassium fertilization treatments, the soil in the experimental plots was homogenized to avoid localized potassium nutrient imbalances. During the potassium treatment, the total irrigation amount during the potato entire growing season was 120 mm/667 m², and other cultivation and field management practices followed the same methods as local farmers. Sampling was collected at 45 days after potato emergence (during the tuber bulking stage). The sampling time was chosen in the morning (8:00-10:00), preferably on clear, windless, and rain-free days, in order to avoid the effects of midday sun exposure, which could lead to photorespiration or photoinhibition in potatoes. The samples were divided into five types of tissue: roots, stems, leaves, tubers, and flowers. Each tissue was rapidly frozen in liquid nitrogen. Roots were cleaned with distilled water to remove any soil before freezing.

### 2.8 Transcriptome sequencing analysis of *StHAK* genes expression levels

Under field experimental conditions, samples from tissues (roots, leaves, tubers) and potassium fertilizer treatments (0, 300, 600 kg/hm^2^) were collected at 50 days after potato emergence (tuber expansion stage). Total RNA was extracted from these samples using the RNAprep Pure Plant Kit (Tiangen, Beijing, China) according to the instructions provided by the manufacturer. RNA concentration and purity was assessed using a NanoDrop 2000 spectrophotometer (Thermo Fisher Scientific, Wilmington, DE). RNA integrity was evaluated with the RNA Nano 6000 Assay Kit on the Agilent Bioanalyzer 2100 system (Agilent Technologies, CA, USA). A total of 1 μg RNA per sample was used as input material for RNA sample preparation. Sequencing libraries were generated using Hieff NGS Ultima Dual-mode mRNA Library Prep Kit for Illumina (Yeasen Biotechnology (Shanghai) Co., Ltd.) following manufacturer’s recommendations and index codes were added to attribute sequences to each sample. Briefly, mRNA was purified from total RNA using poly-T oligo-attached magnetic beads. First-strand cDNA synthesis was followed by second-strand cDNA synthesis. Remaining overhangs were converted into blunt ends via exonuclease/polymerase activities. After adenylation of 3’ ends of DNA fragments, NEBNext Adaptor with hairpin loop structure were ligated to prepare for hybridization. The library fragments were purified with AMPure XP system (Beckman Coulter, Beverly, USA). Then 3 μl USER Enzyme (NEB, USA) was used with size-selected, adaptor-ligated cDNA at 37°C for 15 min followed by 5 min at 95°C before PCR. Then PCR was performed with Phusion High-Fidelity DNA polymerase, Universal PCR primers and Index (X) Primer. At last, PCR products were purified (AMPure XP system) and library quality was assessed on the Agilent Bioanalyzer 2100 system. The libraries were sequenced on an Illumina NovaSeq platform to generate 150 bp paired-end reads. The raw reads were further processed with a bioinformatic pipelinetool, BMKCloud (www.biocloud.net) online platform. The gene sequences of DMv6.1 from spud was selected as the reference genome (Xu et al., 2011). Quantification of gene expression levels were estimated by fragments per kilobase of transcript per million fragments mapped (FPKM) (Trapnell et al., 2010). The selection criteria for DEGs were as follows: genes with a Fold Change (FC) ≥1.5 and p-value ≤ 0.01 were considered significantly differentially expressed. After standardizing and quality-controlling the raw data, differential analysis was conducted using edgeR_DESeq2. All these analysis were performed using BMKCloud (www.biocloud.net).

### 2.9 qRT-PCR and expression pattern assay of *StHAKs*

For tissue samples, 45-day-old potato plants after emergence were randomly sampled with three replicates. The samples were divided into five different tissues including roots, stems, leaves, flowers and tubers. Tissue samples were clipped with DEPC water-socked scissors, and roots and tubers were carefully washed with distilled water to clean the soil. All tissues were immediately stored into liquid nitrogen. For the potassium fertilizer treatment experiment, three randomly selected potato leaves (the fourth from the top) were collected at each potassium level and rapidly frozen into liquid nitrogen.

Real-time PCR assays were conducted according to our previous studies with minor modifications (Liu et al., 2019). Total RNA of each samples was extracted via a total RNA extraction kit (Tiangen, China). Quality evaluation of RNA was done by agarose gel electrophoresis and Quawell micro volume spectrophotometer (Q5000, USA), with the concentrations of total RNA ranging from 200 to 400 ng/μL and A260/280 ratios between 2.0~2.1. Then, 1μg total RNA was digested by DNase I and reverse-transcribed into cDNA based on TransScript gDNA Removal and cDNA Synthesis SuperMix Kit (TransGen, Beijing, China, Cat# AT311). The qRT-PCR was carried out through the QuantiNova SYBR Green PCR kit (QIAGEN, Germany) on a LightCycler 480 system (Roche, Basel, Switzerland). The thermal cycling parameters was set as follows: 95°C for 30 s, followed by 40 cycles of 95°C for 5 s, 60°C for 30 s and 72°C for 15 s. After 40 cycles, the temperature continuous boost from 60 to 90°C and fluorescence signal was continuous collected. At this period, the melting curve was analyzed to access the purity of amplified product. The qRT-PCR experiment for different tissues and potassium treatments were preformed with three technical replicates. The expression level of *StHAK* genes were calculated through 2^-ΔΔCt^ method and *StActin* was chosen as internal reference gene in real time PCR assay (Livak and Schmittgen, 2001). All primers used in this study are listed in (**Supplementary Table 2**).

## 3 Results

### 3.1 Identification of the *StHAKs* in potato

Based on the characteristics of the ‘K-trans’ domain (PF02705), which is specific to the *HAK/KUP/KT* potassium transporter family, a total of 24 *StHAK* genes were identified in potato (*S. tuberosum*) (**Table 1**). After comparing with Phytozome and Spud databases, redundant candidates with low visibility and incomplete domains were excluded. The result showed that all *HAK* genes were unevenly distributed on ten chromosomes. Chromosome 1, 3, 8 and 10 each contained only one gene, chromosome 5 and 6 contained two genes, chromosome 2 and 9 contained three genes, chromosome 4 and 12 contained five genes. Notably, no *StHAK* genes were localized on chromosome 7 and 11.

**Table 1:** Overview and identification of *StHAKs* in *Solanum tuberosum*.

Gene Name	Gene ID	Chromosome location	length of protein (aa)	length of CDS (nt)	length of cDNA (nt)	length of genomic (nt)	Exon	Intron	TMS	pI	Relative molecular mass (Da)	Consevred domains	Subcellular localization
*StHAK1*	***Soltu.DM.01G044390.1***	chr01:82373947 - 82384081	849	2550	3411	10135	10	9	12	5.92	94549.18	PLN00151	plas
*StHAK2*	***Soltu.DM.02G003540.1***	chr02:14787652 - 14799207	784	2355	3005	11556	8	7	12	7.29	88145.59	K_trans super family	plas
*StHAK3*	***Soltu.DM.02G011430.1***	chr02:26177425 - 26166795	792	2379	4247	10631	8	7	12	8.36	89079.77	K_trans super family	plas
*StHAK4*	***Soltu.DM.02G032740.1***	chr02:44416559 - 44410322	772	2319	3142	6238	8	7	12	8.03	87023.98	K_trans super family	cyto
*StHAK5*	***Soltu.DM.03G019580.1***	chr03:44298265 - 44293806	778	2337	2337	4460	10	9	12	9.12	87240.25	K_trans super family	plas
*StHAK6*	***Soltu.DM.04G004120.1***	chr04:4453975 - 4448012	692	2079	2660	5964	8	7	12	8.69	76968.21	K_trans super family	plas
*StHAK7*	***Soltu.DM.04G004170.1***	chr04:4478317 - 4472171	710	2133	2877	6147	8	7	12	8.66	79013.64	K_trans	plas
*StHAK8*	***Soltu.DM.04G004220.1***	chr04:4515833 - 4509523	511	1536	2659	6311	8	7	12	8.57	56069.93	K_trans super family	vacu
*StHAK9*	***Soltu.DM.04G012990.1***	chr04:16253485 - 16247272	779	2340	2756	6214	8	7	12	8.78	87173.01	K_trans super family	cyto
*StHAK10*	***Soltu.DM.04G013040.1***	chr04:16511287 - 16504415	848	2547	2942	6837	9	8	12	6.59	93979.8	PLN00151	plas
*StHAK11*	***Soltu.DM.05G008070.1***	chr05:7978286 - 7972520	779	2340	2745	6150	8	7	12	8.56	87104.18	PLN00149	plas
*StHAK12*	***Soltu.DM.05G008080.1***	chr05:8004302 - 7993602	1205	3618	4048	10701	18	17	12	8.12	134137.89	PLN00151, Rrp15p	plas
*StHAK13*	***Soltu.DM.06G010960.1***	chr06:32838931 - 32831165	790	2373	2922	7767	9	8	12	7.34	87899.87	K_trans super family	plas
*StHAK14*	***Soltu.DM.06G013620.1***	chr06:38566152 - 38562305	533	1602	1602	3848	6	5	8	8.89	59894.51	K_trans super family	plas
*StHAK15*	***Soltu.DM.08G006250.1***	chr08:8853783 - 8847648	817	2454	3051	6136	9	8	12	9.05	91021.82	K_trans super family	plas
*StHAK16*	***Soltu.DM.09G023160.1***	chr09:59036109 - 59030269	657	1974	1974	5841	7	6	10	9.03	74695.89	K_trans super family	plas
*StHAK17*	***Soltu.DM.09G023170.1***	chr09:59056051 - 59051767	692	2079	2079	4285	8	7	6	8.93	78258.83	K_trans super family	plas
*StHAK18*	***Soltu.DM.09G023190.1***	chr09:59073997 - 59070993	644	1935	1935	3005	7	6	10	8.49	72447.31	K_trans super family	plas
*StHAK19*	***Soltu.DM.10G012770.1***	chr10:36777496 - 36797574	863	2592	2592	20079	11	10	12	6	95893.58	PLN00151	plas
*StHAK20*	***Soltu.DM.12G003840.1***	chr12:3128316 - 3121095	894	2685	2923	7222	11	10	12	8.97	99347.05	K_trans super family	plas
*StHAK21*	***Soltu.DM.12G020180.1***	chr12:48229263 - 48237952	804	2415	2870	8690	9	8	12	8.9	89083.4	K_trans super family	plas
*StHAK22*	***Soltu.DM.12G023000.1***	chr12:52885688 - 52890868	784	2355	2565	5181	8	7	12	8.86	88240.53	K_trans super family	plas
*StHAK23*	***Soltu.DM.12G023010.1***	chr12:52893672 - 52897211	376	1131	1131	3540	7	6	7	9.04	41492.4	K_trans super family	plas
*StHAK24*	***Soltu.DM.12G023020.1***	chr12:52897600 - 52898868	422	1269	1269	1269	1	0	4	6.68	48181.69	K_trans super family	chlo,nucl,plas

plas, plasma membrane; vacu, vacuole; cyto, cytoplasm; chlo, chloroplast; nucl, nucleus; Isoelectric point (pI) and Molecular weight (Mw) were predicted by ExPASy (https://web.expasy.org/compute_pi/); Transmembrane domains (TMS) possessed by StHAKs, as predicted by TMHHM Server v.2.0 (http://www.cbs.dtu.dk/services/TMHMM/); Consevred domains of StHAK proteins were investigated using the NCBI Conserved Domain Database (https://www.ncbi.nlm.nih.gov/Structure/bwrpsb/bwrpsb.cgi); Subsellular localization of all StHAK proteins were predicted by WOLF PSORT (https://www.genscript.com/wolf-psort.html).

All genes were named based on their order of location on chromosomes, and all 24 *StHAKs* possess the typical “K-trans” domain. The amino acid lengths of these genes ranged from 422 to 1205, with an average length of 734. *StHAK12* had the longest amino acids sequences, while *StHAK24* had the shortest. The theoretical isoelectric point (pI) and relative molecular mass (Da) of these genes varied from 5.92 to 9.12 (with an average of 8.20) and 41.49 to 134.14 kDa (with an average of 82.79 kDa), respectively. The number of transmembrane domains (TMS) ranges from 4 to 12, with most HAK proteins containing 12 transmembrane helices. Except for *StHAK24*, all genes contained multiple exons and introns. The subcellular location of StHAKs predicted by WoLF PSORT indicated that most protein were located in the plasma membrane, which were consistent with their role in maintaining K^+^ homeostasis in potato. However, StHAK4 and StHAK9 were predicated to be localized in the cytoplasm, and StHAK8 was located in vacuole (**Table 1**).

### 3.2 Classification and phylogenetic analysis of StHAKs in potato

**Figure 1:**
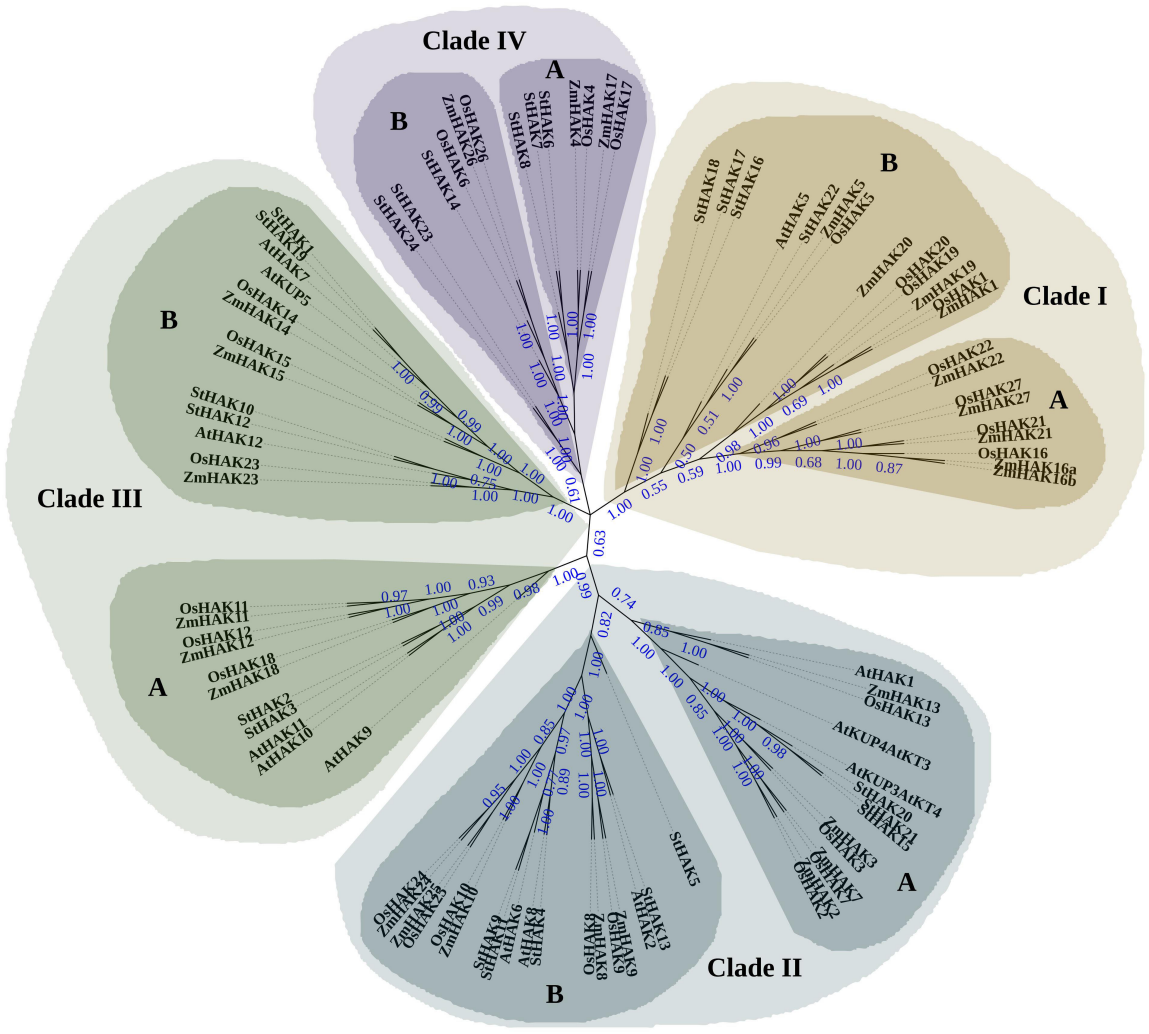
Phylogenetic analysis of HAK/KUP/KT family proteins from potato, rice, maize and *Arabidopsis*. The purple dots on the branches represent bootstrap values. Higher bootstrap values indicated a relatively higher confidence level for the corresponding branch. The full-length protein sequences of 13 KUPs from *Arabidopsis*, 27 HAKs from rice, 27 HAKs from maize, and 24 HAKs from potato were aligned using ClustalW. A phylogenetic tree was subsequently constructed using the neighbor-joining method (NJ) with MEGA7.0. The StHAKs could be grouped into four distinct clusters and were indicated with different colors, consistent with the classification criteria for HAK transporters in maize, rice, and *Arabidopsis*. Each cluster was further divided into two sub-clusters, designated as A and B. St, *Solanum tuberosum*; Os, *Oryza sativa*; Zm, *Zea mays*; At, *Arabidopsis thaliana*.

To explore the evolutionary relationships of StHAKs in potato, the full-length protein sequences of 13 KUPs in *Arabidopsis* (MaüSer et al., 2001), 27 HAKs in rice (Gupta et al., 2008), 27 HAKs in maize (Zhang et al., 2012), 24 HAKs in potato were aligned with Cluster W. A phylogenetic tree was then constructed via the neighbor-joining method and maximum likelihood method (**Figure 1** and **Supplementary Data sheet 1**). The trees showed that the StHAKs could be categorized into four distinct clusters based on the classification criteria of HAK transporters in maize, rice and *Arabidopsis.* Besides, these clusters were further subdivided into sub-cluster A and B (**Figure 1** and **Supplementary Data sheet 1**).

StHAKs from closely related species tended to clustered together. Clade I contained 22 transporters, including 9 from maize, 8 from rice, 1 from *Arabidopsis* (AtHAK5) and 4 from potato (StHAK16, StHAK17, StHAK18, StHAK22). Clade II consisted of 32 transporters, with 9 each from maize and rice, 6 from *Arabidopsis* and 8 from potato (StHAK4, StHAK5, StHAK9, StHAK11, StHAK13, StHAK15, StHAK20, StHAK21). 24 transporters were categorized into Clade III, with 6 from maize, rice and *Arabidopsis*, and potato (StHAK1, StHAK2, StHAK3, StHAK10, StHAK12, StHAK19), respectively. Clade IV had the fewest StHAKs, with 3 from maize, 4 from rice and 6 from potato (StHAK6, StHAK7, StHAK8, StHAK14, StHAK23, StHAK24). No StHAKs were belonged in Clade IA.

### 3.3 Gene structures, conserved motifs and domains analysis in *StHAKs*

To investigate the gene structures, conserved motifs and domains of *StHAKs*, detailed information on the domains of the 24 StHAK protein sequences was assayed using SMART, HMMER, and CDD database, as shown in **Table 1**. The results predicted by SMART and HMMER were largely consistent, while those from CDD database showed some discrepancies. Most StHAK proteins contained only a single ‘K_trans’ domain, but two ‘K_trans’ domain were identified in StHAK12, StHAK14, StHAK17 and StHAK18 via the SMART and HMMER tools. In contrast, the CDD database predominantly identified a single ‘K_trans superfamily’ domain in most StHAK proteins, except for StHAK12, which contained both ‘K_trans superfamily’ and ‘Rrp15p’ domains. The presence of similar domains suggested that the HAK family members with the same domain may belong to the same subfamily, for instance, StHAK16, StHAK17 and StHAK18, all of which belonged to Clade IB, indicating that the protein structure was conserved within this specific subfamily.

**Figure 2:**
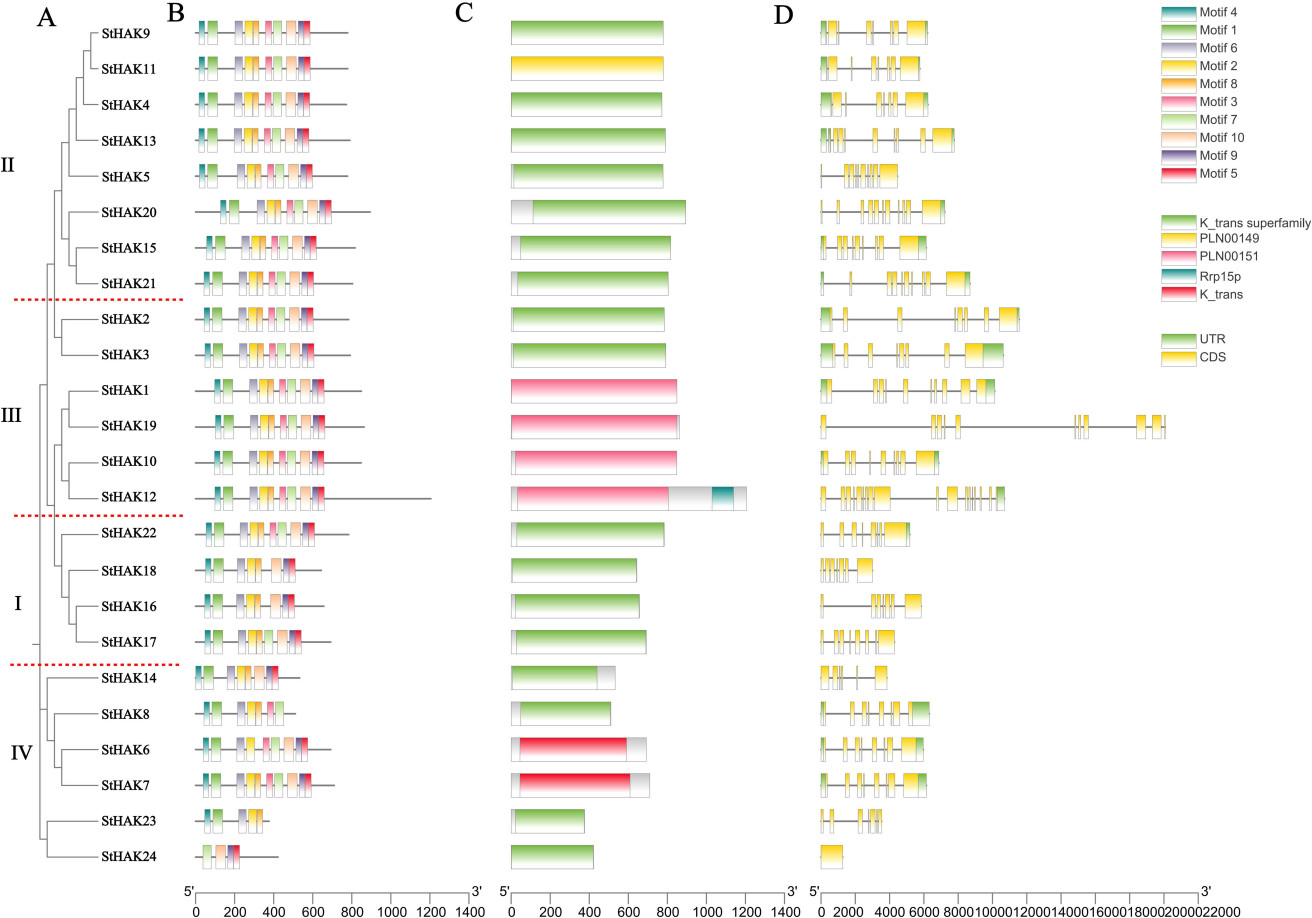
Phylogenetic relationships, conserved motifs and gene structures of the *StHAKs*. **(A)** The phylogenetic tree was constructed according to the full-length sequences of 24 potato HAK/KUP/KT family proteins. The different Clades were separated by red dashed lines. **(B)** The conserved motifs of StHAK proteins. The gray horizontal lines represented the amino acid lengths of the sequences, and the different colors on the sequences denoted various motif types. **(C)** The domain of StHAK proteins. The gray regions indicated areas without specific domains, while the colored sections marked the locations of the K_trans domain. **(D)** The Exon-intron structures of *StHAK* genes. UTR, untranslated region. the gray horizontal lines represented intron regions, green corresponded to UTRs, and yellow corresponded to CDS regions. At the bottom of the figures, “5′ “ and “3′ “ indicated direction of the sequences, while the numbers represented the length of the sequences in amino acids. The legend comprised three parts: the top section used different colors to represent various motif types, the middle section indicated different domain types, and the bottom section showed the positions of the UTR and CDS regions.

In this study, 10 distinct conserved motifs were identified in the StHAKs via the MEME online program, and they were labeled as motifs 1 to 10. The sequence and logos for these conserved motifs and their distribution in each StHAK protein were illustrated in **Figure 2** and **Supplementary Tables 2**, **3**. Generally, motif 1, 2, 4 and 6 were found in nearly all StHAKs, with the exception of StHAK24. StHAK24, which belonged to Clade IB, had the fewest motifs, containing only 4 motifs (**Figures 2A**, **B**). Similarly, StHAK23, another member of Clade I, exhibited a motif distribution similar to StHAK24, possessing only 5 motifs. The fewer conserved motifs in StHAK23 and StHAK24 may be related to their shorter lengths of protein (**Table 1**). In contrast, the number of conserved motifs in Clade II and Clade III were consistent, with all 10 conserved motifs were present in these subfamilies, suggesting potential functional similarities among these StHAKs. Within Clade IV, StHAK6, StHAK8 and StHAK14 contained 9, 7 and 8 conserved motifs, respectively, while all motifs were present in StHAK7 (**Figure 2B**). The variation in motif categories among different subfamilies may reflect the conservation and unique functions of the HAK protein family. The presence and arrangement of certain motifs within the same subfamily indicated that the structure of StHAKs is both conserved and distinctive within specific subfamilies.

Conserved domain prediction results revealed that all StHAKs contain the typical ‘K_trans’ domains within their sequences, confirming their classification as members of the HAK/KUP/KT gene family (**Figure 2C**). To investigate the structural characteristics of the StHAKs, we analyzed the gene structure of the 24 StHAKs using GSDS2.0, mapping the exon/intron organization alongside conserved motifs and a phylogenetic tree (**Figure 2D**). The number of exon and intron varied across the four Clades, with Clade III exhibiting a higher exon/intron count compared to the other three Clades (**Figure 2D**; **Supplementary Table 4**). Notably, StHAK12, a member of Clade III, had the most exons, totaling 18. The exon number in Clade IV ranged from 1 to 8, with an average of 6.3, which is lower than in the other Clades. Interestingly, StHAK24, a Clade IV member, harbored only one exon and no intron, which with the fewest exon in the entire *HAK* gene family (**Supplementary Table 4**). These findings suggested that a reconstruction of gene structure occurred during the evolutionary divergence of altered HAKs subfamilies in potato.

### 3.4 Chromosomal location, collinearity and synteny analysis of *StHAKs* in potato

**Figure 3:**
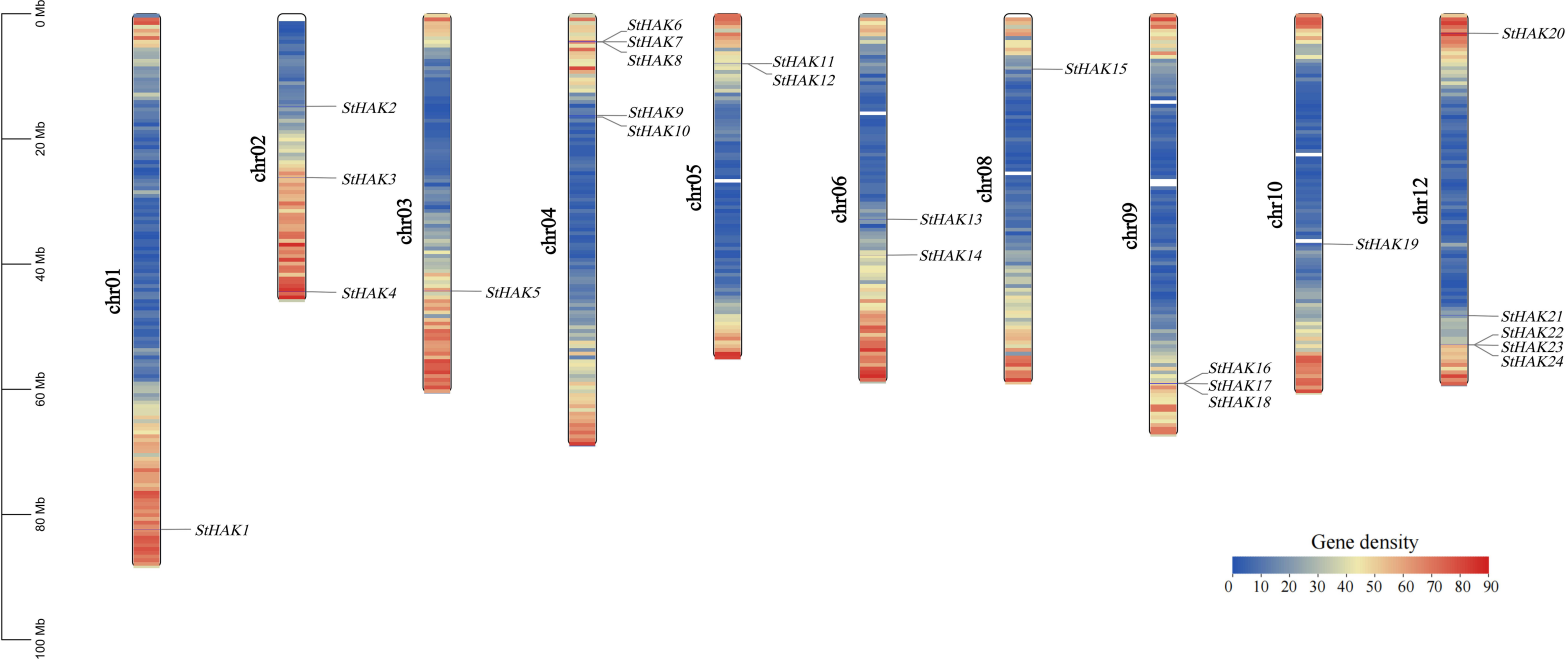
The Chromosomal localization of the *StHAK* genes. The black lines indicated the position of each *StHAK* genes. Different colors within the chromosomes represented the gene density. The legend on the left indicated the size of the chromosomes. From left to right, the chromosomes were arranged in order from 1 to 12, except for chromosomes 7 and 11. The white regions within the chromosomes represented areas without corresponding gene information. In the gene density color key, the numbers indicated the number of genes.

The *StHAKs* were mapped onto chromosomes based on the potato reference genome (Potato Genomics Resource). The *StHAK* genes were generally distributed across the genome, while their distribution across the chromosomes is uneven (**Figure 3**). Five *StHAK* genes (20.83%) were located on both chromosomes 4 and 12, which is the highest concentration among the ten chromosomes. In contrast, only one gene (4.17%) was found on chromosomes 1, 3, 8 and 10 were distributed with two (8.33%) on chromosomes 5 and 6, and three (12.5%) on chromosomes 2 and 9. However, no *HAK* genes were distributed on chromosomes 7 and 11.

Gene density varied across different regions of genomes. In the *StHAK* genes family, gene density profiles were assayed via Gene Destiny Profile of TBtools. Numerous *StHAK* genes were unevenly distributed on chromosomes, with a higher destiny observed at the ends of the long and short arms and a lower destiny in the mid-chromosomal regions (**Figure 3**). The majority of *StHAK* genes family members were located in the terminal regions of the chromosomes. The color shift from blue to red on the chromosomes maps indicated an accumulation of genes, whereas blank areas represent regions with lower gene density.

**Figure 4:**
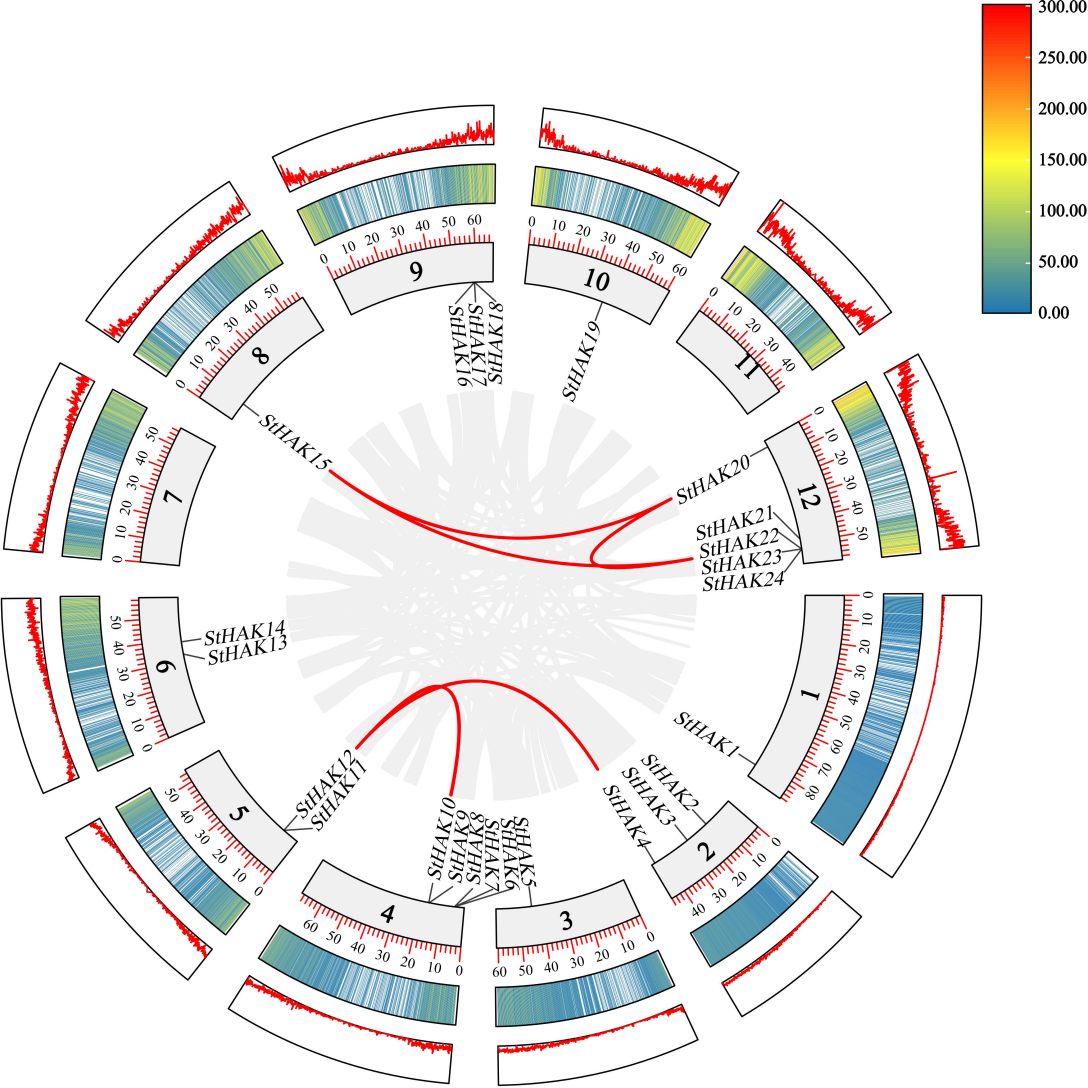
Collinearity analysis of *HAK* genes in potato. Collinearity refers to the conserved order of genes along a chromosome. It emphasizes the sequential arrangement of homologous genes, often focusing on their relative positions and orientation across chromosomes within or between species. It is often used to analyze evolutionary events like genome duplications or rearrangements. The grey lines on the inside of the ring represented all collinear blocks within the potato genome. The red lines on the inside of the ring indicated the duplicated HAK gene pairs in potato. The two rings on the outside of the chromosome ring represented gene density, with different colors indicating different gene densities. The numbers in the color key at the top right represented the number of genes.

**Figure 5:**
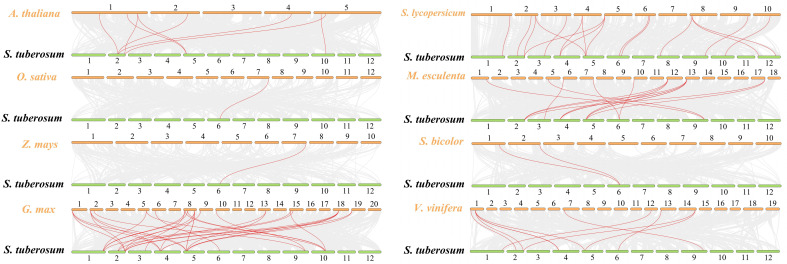
Synteny analyses between *HAK* genes of potato and other eight species (*Arabidopsis thaliana*, *Oryza sativa*, *Zea mays*, *Glycine max*, *Solanum lycopersicum*, *Manihot esculenta*, *Sorghum bicolor*, *Vitis vinifera*). Synteny refers to the co-localization of genes on the same chromosome, without necessarily preserving their order or orientation. Synteny simply indicates that a set of genes appears together on the same chromosome in different species or genomes, regardless of the sequence. Small green rectangles represented potato chromosomes, while small orange rectangles represented chromosomes from eight species. Grey lines indicated synteny blocks between potato and other plant genomes, while red lines highlighted syntenic *HAK* gene pairs. The number of syntenic gene pairs between potato and other species are as follows: *Arabidopsis thaliana* (8), rice (1), maize (1), soybean (33), tomato (17), cassava (19), sorghum (2), and grape (10).

Collinear gene pairs within the potato genome were analyzed to elucidate the relationship among the *HAK* genes and other potential gene duplication events (**Figure 4**). The results of collinearity analysis revealed that six genes pairs (*StHAK4/11*, *StHAK9/11*, *StHAK10/12*, S*tHAK15/21*, *StHAK15/20*,*StHAK20/21*) were found in potato genome, and were distributed across different chromosomes. No significant tandem duplication or segmental duplication gene pairs were identified within the potato genome. To further investigate the syntenic relationship of *HAK* gene families between potato and other species, syntenic analyses were conducted with eight species, including three monocots (*O.Sativa* and *S. bicolor*, *Z.mays*) and five dicots (*A.thaliana*, *V.vinifera*, *G.max*, *M.esculenta*, *S. lycopersicum*) (**Figure 5**; **Supplementary Table 5**). A total of 91 orthologous gene pairs were identified. The analysis showed that 33 gene pairs were found between *G.max* and *S.tuberosum*, followed by *M.esculenta* (19), *S. lycopersicum* (17), *V.vinifera* (10)*, A.thaliana* (8), *S. bicolor* (2), *O.Sativa* (1), *Z.mays* (1). In comparison with dicotyledonous plants, fewer collinear relationships were detected between *S.tuberosum* and monocotyledonous plants like *O.Sativa* and *Z.mays*. The fewest collinear pairs were found on chromosomes 8 and 12, while the highest number were observed on chromosomes 2 and 6, and the distribution of these collinear pairs on chromosome 6 were rather equally across species. Interestingly, the collinear relationships of monocots were all identified on chromosome 6, suggesting that these orthologous pairs may have existed prior to the ancestral divergence.

### 3.5 Analysis of promoter cis-acting elements of *StHAKs* in potato

**Figure 6:**
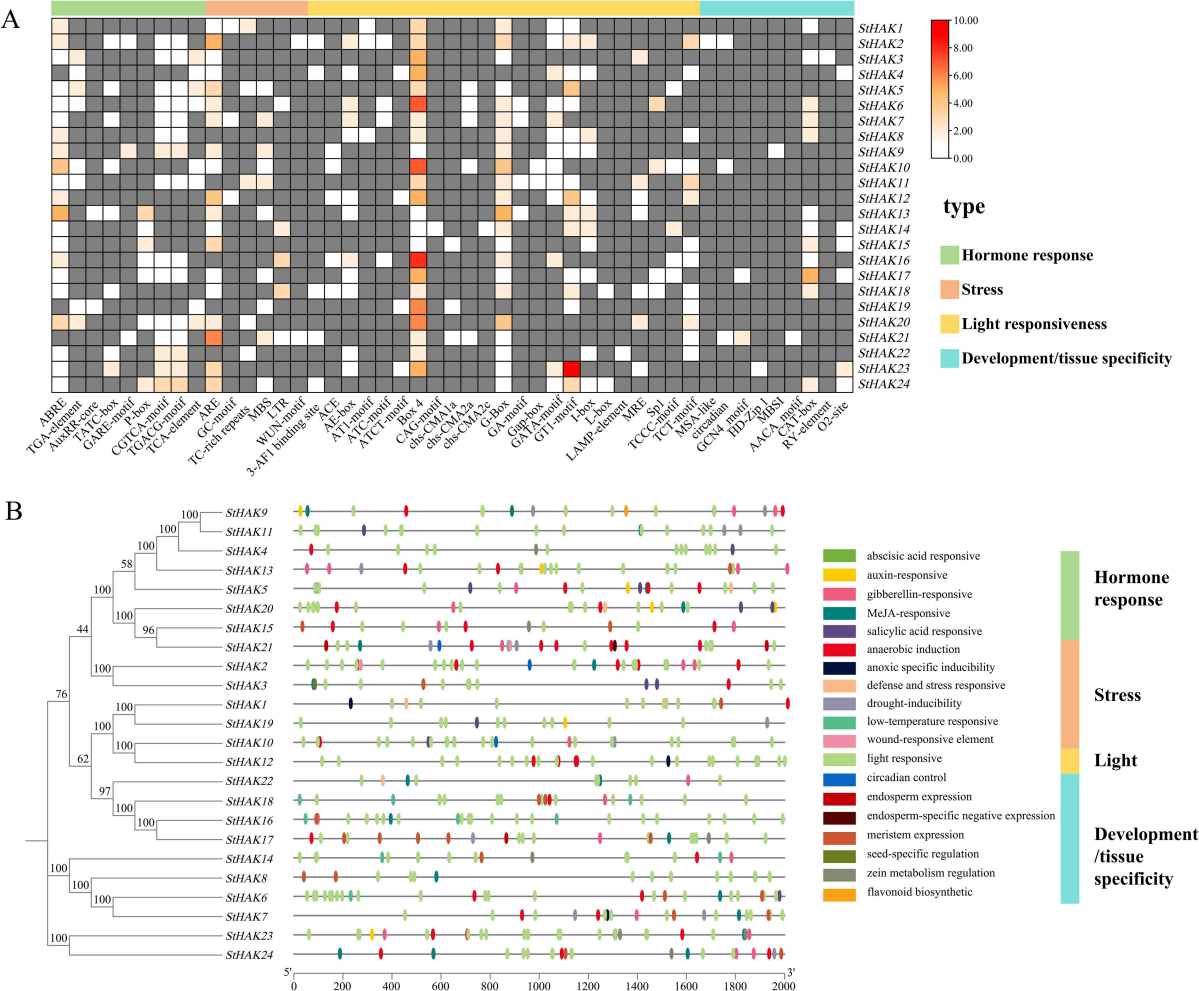
Analysis of cis-acting elements in *StHAKs*. Analysis of cis-acting elements was conducted on 2 kb sequences upstream of coding sequences of *StHAKs*. **(A)** Classification and number of cis-acting elements on the promoter region of *StHAKs*. The promoter regions of the *StHAKs* genes were primarily divided into four major categories of elements: light green represents hormone response-related elements, light pink represents stress response-related elements, yellow represents light response-related elements, and blue represents development and tissue-specific elements. The small boxes within the large rectangles indicated the number of these elements in the *StHAKs* genes, with a gradient from white to red signifying an increasing number of elements. Gray indicated the absence of these elements in the *StHAKs* genes. The color key on the top right referred to the number of cis-elements. **(B)** Distribution of cis-acting elements in promoter region of *StHAKs*. The phylogenetic tree was constructed according to the full-length sequences of 24 potato HAK/KUP/KT family proteins, which were presented in left. On the right side, the selected promoter lengths (2000 bp) were displayed, along with the cis-acting elements located at different positions within the promoters, represented by small ovals in different colors.

The cis-acting elements of the *StHAK* genes were analyzed within the 2000-bp upstream regulatory region from the start codon ATG. A total of 533 cis-elements were identified in the promoter regions of the *StHAK* genes and mainly four groups were categorized: hormone responsive (118, 22.1%), stress (80, 15.0%), light (295, 55.4%) and development/tissue specificity (40, 7.5%) (**Figure 6**; **Supplementary Table 6**). Plenty of phytohormone-responsive elements were present in the promoter regions of the *StHAK* genes, including abscisic acid responsive (ABA) (36, 30.5%), auxin-responsive (IAA) (7, 5.9%), gibberellin-responsive (GA) (24, 20.3%), methyl jasmonate-responsive (MeJA) (40, 33.9%), salicylic acid responsive (SA) (11, 9.3%) (**Figure 6A**; **Supplementary Table 6**). Stress related cis-regulatory elements, such as anaerobic induction (44, 55%), anoxic specific inducibility (3. 3.7%), defense and stress responsive (7, 8.7%), drought-inducibility (14. 17.5%), low-temperature responsive (10. 12.5%) and wound-responsive element (2, 2.5%), were also detected in the *StHAKs* promoter regions (**Figure 6A**; **Supplementary Table 6**). The number of light-responsive elements in the *StHAKs* promoter regions was the highest (295), accounting for nearly 56% of the total, and these elements were distributed almost all *StHAKs* gene promoter regions (**Figure 6B**). Elements related to plant development and tissue specificity, such as meristem expression (22, 55%), zein metabolism regulation (7, 17.5%), endosperm expression (4, 10%), seed specific (1, 2.5%) and palisade mesophyll cells differentiation (1, 2.5%), were also found in the promoter regions of the *StHAK* genes. What’s more, elements involved in cell cycle regulation (1, 2.5%) and circadian control (3, 7.5%) were categorized as development/tissue specificity elements. Overall, these results suggested that multiple cis-acting elements may involve in regulation of *StHAK* genes in response to growth, hormone induction, and abiotic stress.

### 3.6 Array of expression profiles of *StHAKs* based on microarray data

**Figure 7:**
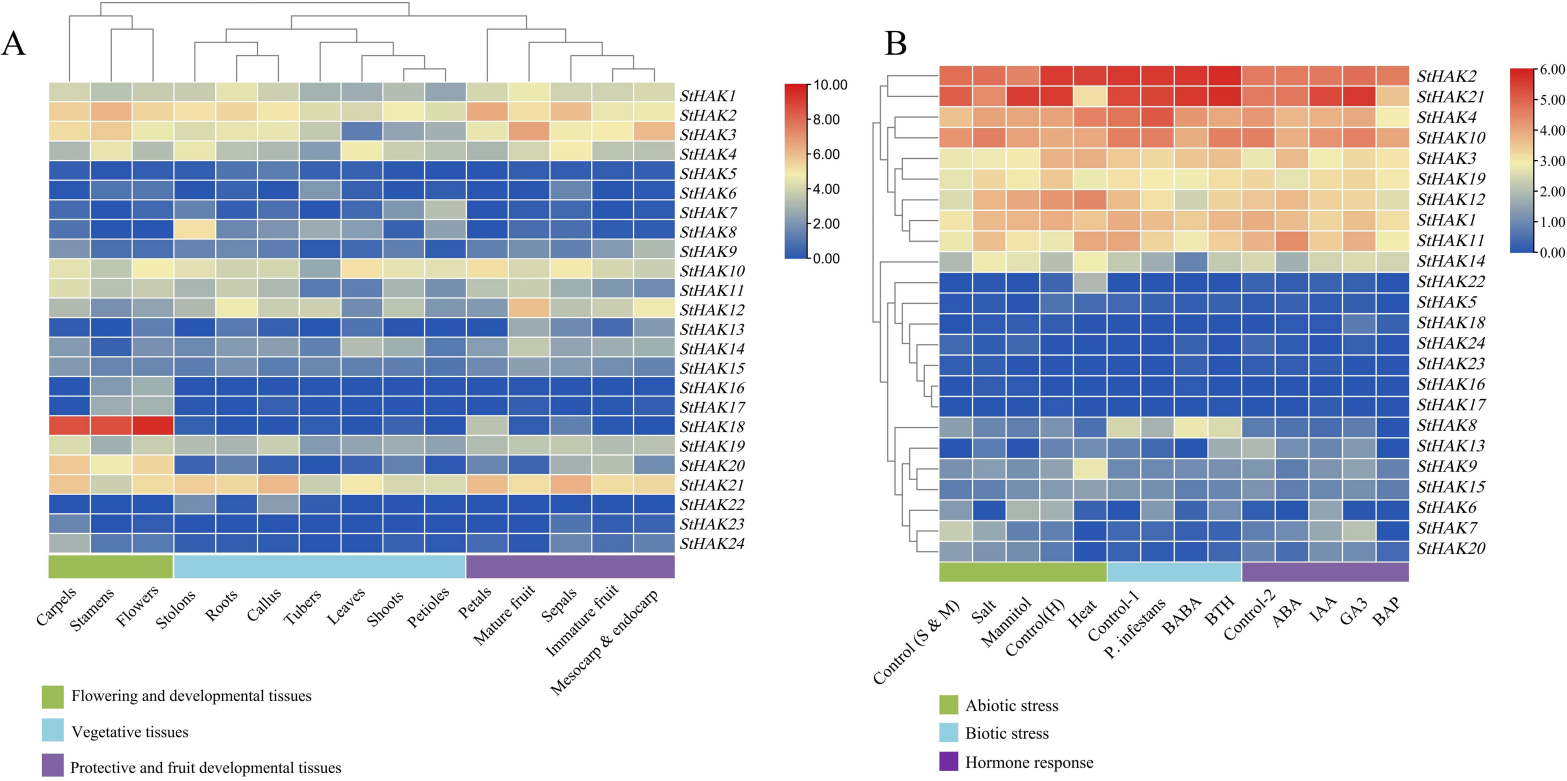
Expression profiling of *StHAKs* based on Microarray data. **(A)** The mircoarray data was download from Potato Genomics Resource (Spud) and the expression level of *StHAKs* in 15 various tissues were analyzed. We performed clustering on tissues with similar *StHAKs* expression levels, categorizing the 15 tissues into three major groups. Green boxes represented flowering and developmental tissues (including carpels, stamens and flowers), blue boxes represented vegetative tissues (including stolons, roots, callus, tubers, leaves, shoots and petioles), and purple boxes represented protective and fruit developmental tissues (petals, mature fruits, sepals, immature fruits, mesocarp & endocarp). The color key indicated the gene expression levels (TPM values), with colors ranging from blue to red, where blue represented lower expression and red represented higher expression. **(B)** Expression levels of *StHAKs* under three types of abiotic stresses, three types of biotic stresses and four types of phytohormone treatments. Control (S & M) referred to the control treatment for salt stress and mannitol stress. Control (H) referred to the control treatment for heat stress. Control-1 referred to the control for biotic stress, and control-2 referred to the control for different hormone treatments. The color key on the top right indicated the gene expression levels (TPM values).The TPM values were normalized by log2 (TPM) transformation.

To investigate the expression level of *StHAKs* in potatoes, their expression patterns were analyzed across various tissues, stress and hormone-induced treatments by means of Microarray data downloaded from Potato Genomics Resource. According to the RNA-Seq data, the expression levels of all 24 *StHAKs* were further examined and interpreted (**Figure 7**; **Supplementary Table 7**). The expression of *StHAKs* in 15 different tissues were analyzed, including sepals, leaves, roots, shoots, callus, tubers, stolons, petioles, petals, stamens, carpels, flowers, mature fruits, immature fruits, mesocarp & endocarp. The results indicated that the expression of the *StHAKs* were significant difference in these tissues (**Figure 7A**). Notably, the expression levels of *StHAKs* of Clade I and Clade IV were generally lower than other Clades in different tissues, such as *StHAK22*, *StHAK23*, *StHAK24* from Clade I and *StHAK6*, *StHAK7*, *StHAK8* from Clade IV. While *StHAK18*, a member of Clade I, was highly expressed in stamen, carpel and flower and almost no expression in other tissues, indicating apparent tissue specificity (**Figure 7A**). Similarly, *StHAK20*, a Clade II member, displayed the same expression pattern as *StHAK18*. Members of Clade III generally showed higher mRNA transcript levels in different tissues, especially *StHAK2* and *StHAK10*. The expression profiles of *StHAK1*, *StHAK3*, *StHAK12* and *StHAK19*, all belonging to Clade III, were somewhat lower in shoot tissues such as leaves and petioles, but were more highly expressed in carpels, stamens, roots, mature fruits, mesocarp & endocarp (**Figure 7A**). The expression pattern of *StHAK21* (Clade II member) was similar to that of *StHAK2*, which was also highly expressed in sepals, callus and petals.

The RNA transcript levels of *StHAKs* under abiotic and biotic stress were also examined (**Figure 7B**). In comparison with control, the expression of *StHAK1*, *StHAK5*, *StHAK11*, *StHAK12*, *StHAK14*, and *StHAK19* genes was significantly upregulated under salt stress, with some genes showing more than a 2-fold increase. Conversely, the expression level of *StHAK6* was markedly reduced under salt stress. To further explored whether the transcript levels of *StHAK* genes are affected by biotic stress, the expression profiles of *StHAKs* were analyzed under three biotic elicitors: β-aminobutyric acid (BABA), benzothiadiazole (BTH) and pathogen treatment (**Figure 7B**). Under pathogen treatment, the expression levels of most *StHAKs* were reduced, except for *StHAK4*, although the reduction was not significant. Similarly, under BABA treatment, the expression pattern of *StHAKs* resembled that observed after pathogen infection, with most *StHAKs* exhibiting a downward trend in expression. In contrast, under BTH treatment, the expression levels of *StHAK9*, *StHAK13*, *StHAK20*, and *StHAK21* were significantly upregulated (**Figure 7B**).

Phytohormones play a crucial role in plant development and growth, ionic homeostasis and stress response. Expression data for *StHAKs* were also obtained from Spud for several hormonal treatments. *StHAK3* and *StHAK5* were highly expressed under ABA and GA3 treatment, whereas their expression was lower under IAA treatment (**Figure 7B**). Conversely, *StHAK20* and *StHAK21* were highly expressed under IAA and GA3 treatments, but *StHAK20* expression was reduced by approximately half under ABA treatment. The expression of *StHAK21* remained relatively unchanged after ABA treatment. *StHAK7* was induced by ABA, IAA and GA3, but inhibited by BAP treatment. *StHAK6* was particularly sensitive to IAA, with expression levels increasing over 20-fold, but it did not respond to other hormonal treatments. With the exception of *StHAK3* and *StHAK18*, the RNA transcript levels of other genes were significantly lower under BAP treatment (**Figure 7B**), indicating that most *StHAKs* were insensitive to BAP treatment.

### 3.7 Array of expression profiles of *StHAKs* based on transcriptome sequencing data and qRT-PCR

**Figure 8:**
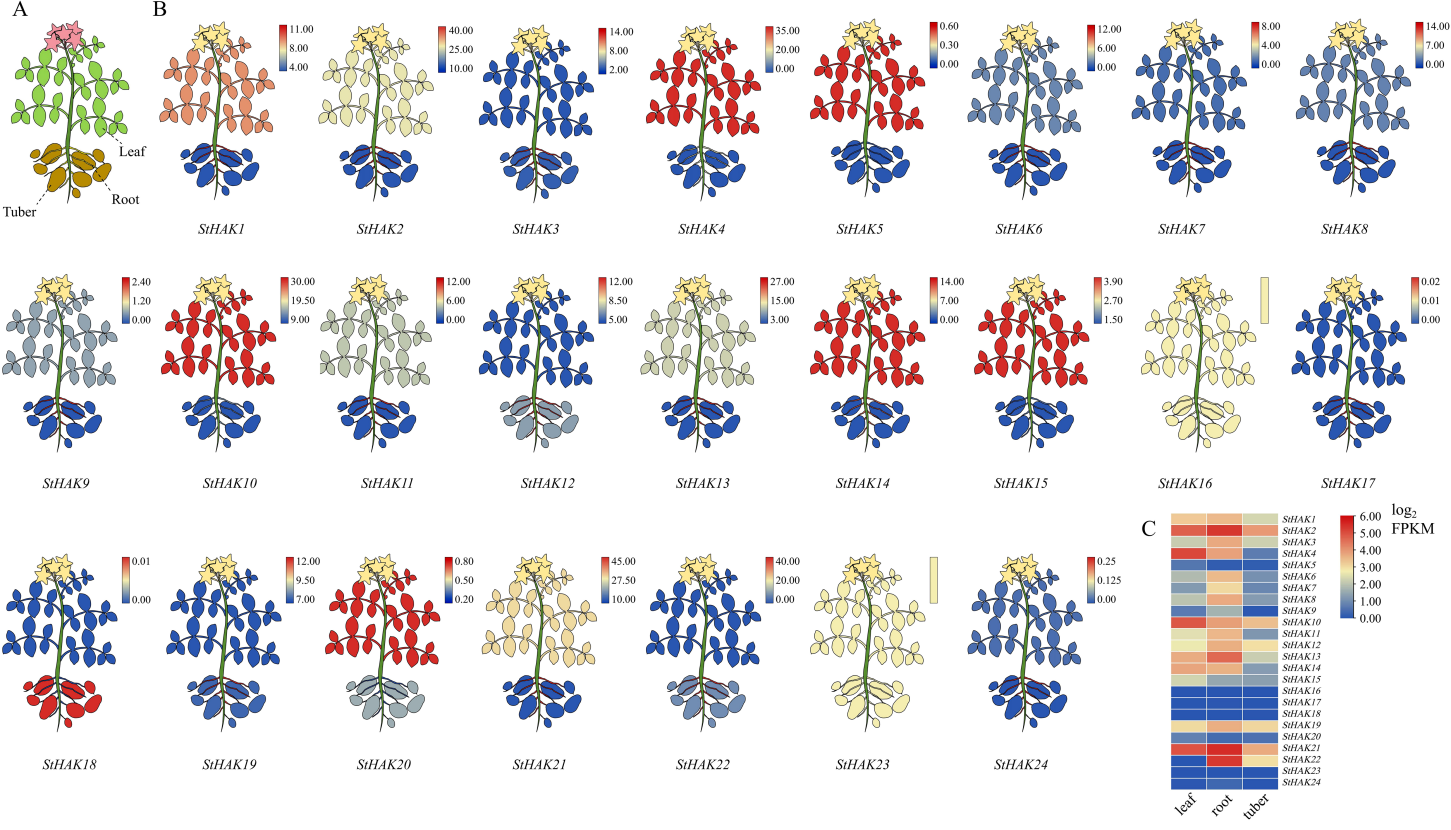
Expression profiling of *StHAKs* based on transcriptomic data. **(A)** Tissues for transcriptome sequencing. The tissues used for transcriptome sequencing mainly included leaf, root and tuber. **(B)** Cartoon plot of *StHAKs* expression in leaf, root and tuber. Different colors represented variations in gene expression levels, ranging from blue to red, with expression levels gradually increasing. The color key indicated the FPKM values of expression levels. The expression levels of *StHAK16* and *StHAK23* were consistently very low across different tissues. **(C)** Heatmap of *StHAKs* expression in leaf, root and tuber. Different colors represented variations in gene expression levels, ranging from blue to red, with expression levels gradually increasing. The FPKM values were normalized by log2 (FPKM) transformation.

To investigate the expression profiles of *StHAKs* in different tissues of potato, three tissues-leaves, roots and tubers-were selected for transcriptome sequencing (**Figure 8A**). The results revealed significant differences in the expression of *StHAKs* in these tissues (**Figure 8B**; **Supplementary Table 8**). In general, the mRNA transcript levels of *StHAK4*, *StHAK5*, *StHAK9* in Clade II, *StHAK24* in Clade I and *StHAK14* in Clade IV were higher in leaves compared to roots and tubers. Among these, *StHAK4* showed the most pronounced variation, which expressed 53.65 times higher in leaves than in tubers, followed by *StHAK9* and *StHAK24*, which expressed 13.22 and 13.14 times higher than in tubers, respectively (**Figure 8C**). Besides, *StHAK5* was 5.32 times more abundant in leaves than in roots. Members of Clade IV were predominantly expressed in roots, with expression levels generally 7-fold higher than in tubers, and *StHAK6* exhibiting the highest increase at 9.57-fold. The mRNA transcript levels of *StHAK4*, *StHAK9*, *StHAK11*, and *StHAK13* in Clade II showed a similar trend, with the expression of *StHAK9* and *StHAK4* in roots were 48.57-fold and 21.20-fold higher than that in tubers, respectively. Notably, *StHAK24* (Clade I) exhibited the most significant increase, with expression in roots 134.43 times higher than in tubers (**Figure 8C**). Overall, the expression of *StHAKs* was lower in tubers compared to leaves and roots, however, *StHAK12* was expressed 1.28 times more in tubers than in leaves, and *StHAK20* was expressed 1.41 times more in tubers than in roots (**Figure 8C**).

**Figure 9:**
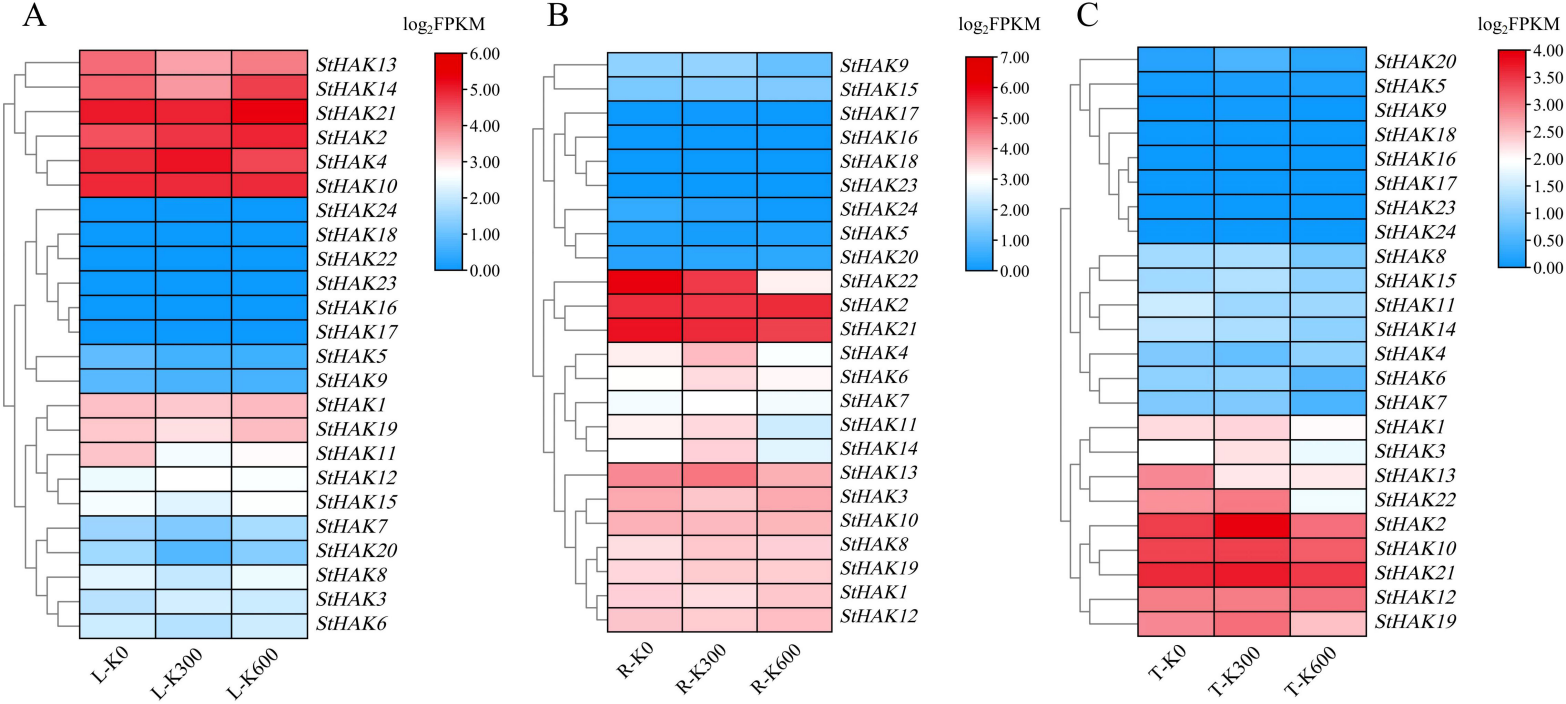
The transcript levels of *StHAKs* in leaf, root and tuber of potato under potassium fertilizer treatment based on transcriptomic data. **(A)** Expression levels of *StHAKs* in leaves. **(B)** Expression levels of *StHAKs* in roots. **(C)** Expression levels of *StHAKs* in tubers. R, roots; L, leaves; T, tubers; K0, 0 kg/hm^2^; K300, 300 kg/hm^2^; K600, 600 kg/hm^2^; Genes with similar expression levels were clustered together. Different colors represented variations in gene expression levels, ranging from blue to red, with expression levels gradually increasing. FPKM values were normalized by log2 (FPKM) transformation.

To explore the response of *StHAKs* to varying potassium concentrations, we established three potassium gradients in the field and sequenced the transcriptomes of roots, stems, and leaves of potato during the tuber expansion stage. The results showed significant differences in the response of *StHAKs* to potassium in different tissues (**Figure 9**; **Supplementary Table 9**). In leaves, as potassium concentration increased, *StHAK2* was upregulated, while the expression of *StHAK3*, *StHAK4* and *StHAK12* was initially increased followed by decreased. In contrast, *StHAK5*, *StHAK9* and *StHAK10* were downregulated. In addition, the mRNA transcript levels of several genes such as *StHAK16*, *StHAK17*, *StHAK18*, *StHAK22*, *StHAK23*, *StHAK24* were extremely low under different doses of potassium (**Figure 9A**). In roots, the expression levels of ten *StHAKs* (*StHAK4*, *StHAK6*, *StHAK7*, *StHAK8*, *StHAK9*, *StHAK11*, *StHAK13*, *StHAK14*, *StHAK15*, *StHAK19*) increased and then decreased with rising potassium concentration, suggesting that a moderate potassium level (300 kg/hm^2^) promotes *StHAKs* expression, while excessive potassium (600 kg/hm^2^) suppressed it. The mRNA transcript levels of *StHAK21*, *StHAK22*, and *StHAK24* gradually declined with increasing potassium concentration, whereas the expression level of *StHAK20* gradually increased. In contrast, *StHAK16*, *StHAK17*, *StHAK18* and *StHAK23* were almost not expressed under varying potassium concentration treatments (**Figure 9B**). In tubers, the expression patterns of *StHAKs* were comparable to those in roots, with eleven genes showing an initial increase followed by a decrease in response to rising potassium levels. Meanwhile, three genes (*StHAK7*, *StHAK13*, *StHAK14*) displayed a decreasing trend, while *StHAK5* and *StHAK12* showed an increasing trend (**Figure 9C**).

**Figure 10:**
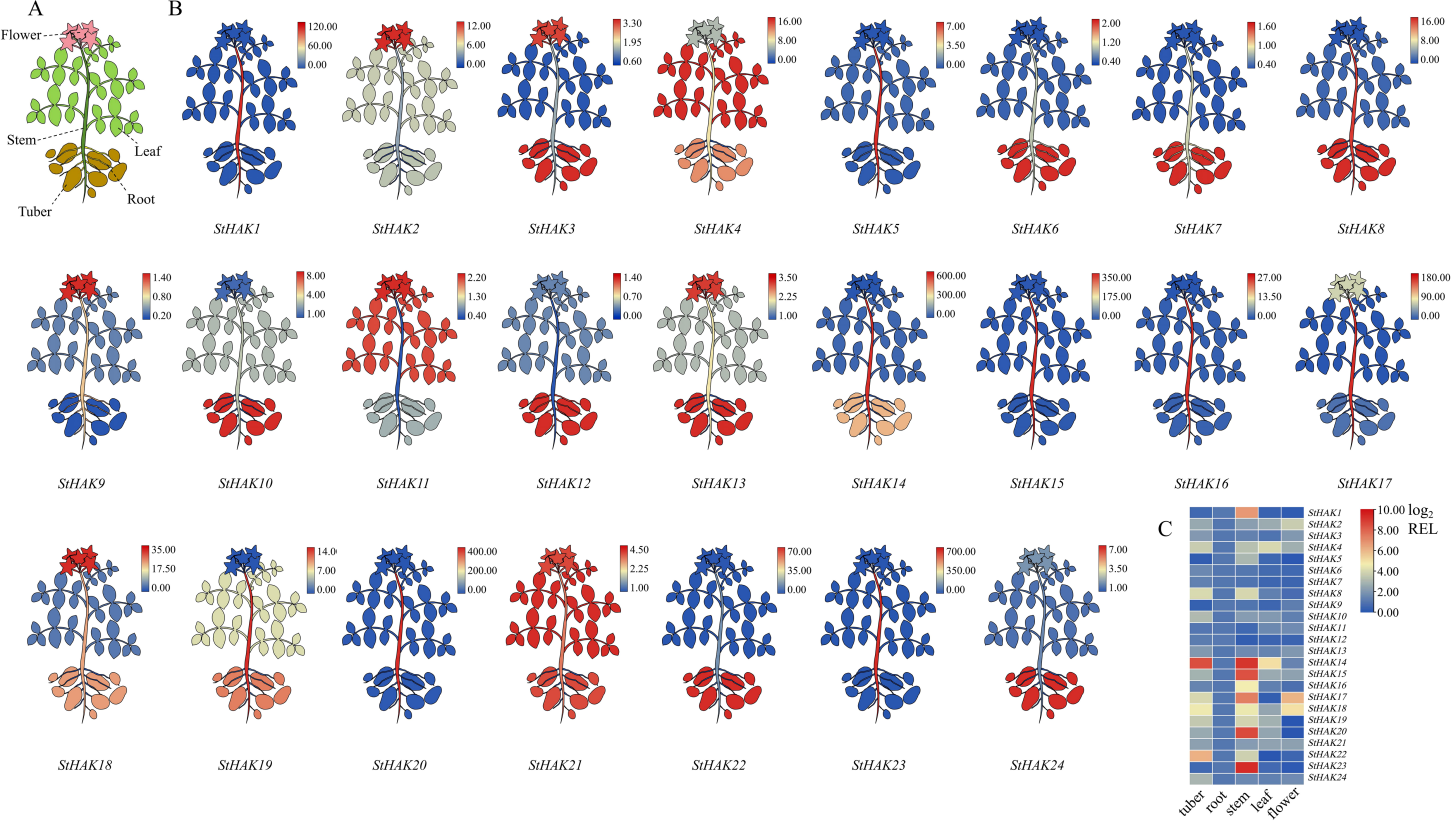
The quantitative real-time-PCR (qRT-PCR) analysis of expression patterns of *StHAKs* at different organs. **(A)** Five tissues for qRT-PCR. The tissues used for qRT-PCR mainly included root, stem, leaf, tuber and flower. **(B)** 24 *StHAKs* were selected for expression level analysis. Different colors represented variations in gene expression levels, ranging from blue to red, with expression levels gradually increasing. The color key indicated the Relative genes expression values of expression levels. **(C)** Heatmap of *StHAK* genes expression in tubers, roots, stems, leaves, flower. The color key indicated the relative genes expression values of expression levels. Relative genes expression values were normalized by log2 (REL) transformation.

To better understand the expression patterns of *StHAKs* in various potato tissues, we selected all genes from each Clade for qRT-PCR analysis. The tissues examined included roots, stems, leaves, tubers and flowers (**Figure 10A**). In short, most of the selected *StHAKs* were expressed at relatively low levels in the roots (**Figures 10B**, **C**). *StHAK8*, *StHAK10*, *StHAK14*, *StHAK19, StHAK22* were highly expressed in stems and tubers, but were poorly expressed in other tissues. Among them, the expression of *StHAK14* in stems and tubers was 560 and 661 times higher than in root, and 320 and 388 times higher than in flowers, respectively (**Figures 10B**, **C**). Many genes showed higher expression in tubers compared to other tissues. In addition to *StHAK4*, there were nine genes, which primarily belonged to Clade IV and Clade III, might play an important role in the growth and development of potato tubers. The transcript levels of *StHAK4* and *StHAK21*, which were all belonged to Clade II, were higher in leaves compared to other tissues, while the expression of *StHAK1*, *StHAK3*, *StHAK8*, *StHAK10*, *StHAK14*, *StHAK17*, *StHAK18*, and *StHAK22* in leaves was significantly lower than in stems and tubers. These results showed that *StHAK4* and *StHAK21* might involved in potato leaf development or in pathways related to the photosynthetic response in leaves. The mRNA levels of *StHAK2*, *StHAK11* and *StHAK18* were higher in flowers, whereas *StHAK1*, *StHAK5*, *StHAK19, StHAK20* and *StHAK23* were expressed at somewhat lower levels in flowers (**Figures 10B**, **C**).

**Figure 11:**
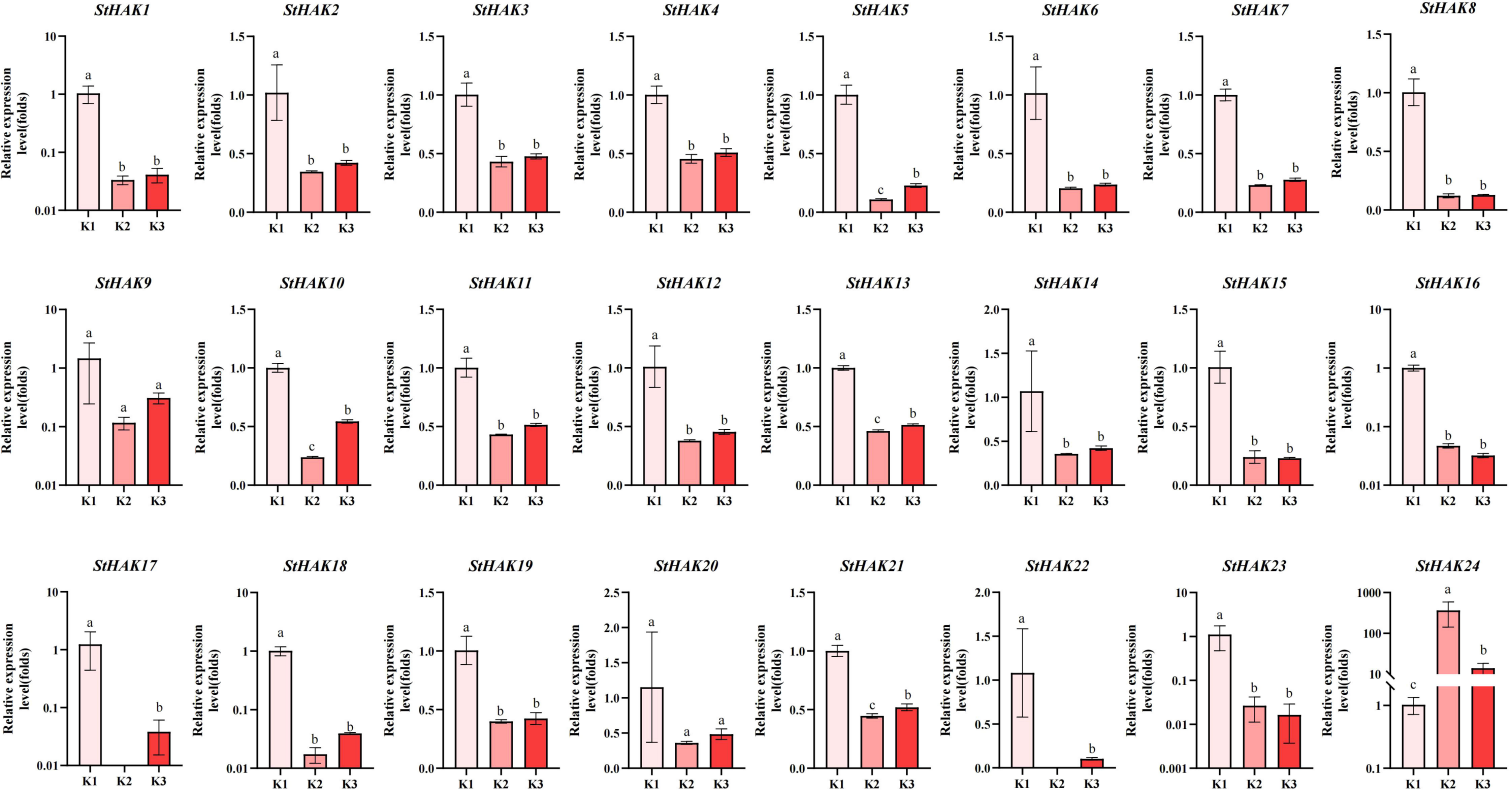
The qRT-PCR analysis of expression patterns of *StHAKs* under different potassium fertilizers. K0, 0 kg/hm^2^; K1, 300 kg/hm^2^; K2, 600 kg/hm^2^; The horizontal axis represented potassium fertilizer treatments, and the vertical axis represented relative gene expression levels. K1 was selected as the control group, while K2 and K3 were used as treatment groups. Vertical bars indicate ± SD (N = 3). Different letters indicated significant differences between treatments (p < 0.05).

The relative abundance levels of *StHAKs* exhibited varying trends under different potassium fertilizer treatments (**Figure 11**). Compared to the K0 treatment, nearly all *StHAK* genes showed a significant decrease in expression under K2 and K3 treatments, which was related to HAK-mediated K^+^ uptake; the expression of *StHAK* genes was significantly upregulated only under very low external potassium concentrations. Interestingly, *StHAK24* displayed a contrasting pattern, with a notable increase in expression under K2 treatment and a decrease under K3 treatment.

## 4 Discussion

Members of the *HAK* gene family have been identified in a broad range of plants, including *Arabidopsis*, rice, wheat, barley, *moso bamboo*, cassava (Gierth et al., 2005; Gupta et al., 2008; Cheng et al., 2018; Cai et al., 2021; Guo et al., 2024; Luo et al., 2024). These genes are involved in the uptake and transport of potassium, as well as in various processes related to plant growth and development (Wang and Wu, 2013). In this study, we identified a total of 24 *StHAKs*, constructed a phylogenetic tree, and analyzed their evolutionary relationships and potential biological functions. What’ more, we examined the gene structure, conserved motifs, chromosomal localization, collinearity, syntenic relationships, and cis-acting elements in the promoter regions of these 24 *StHAKs* to gain a comprehensive understanding of their roles. Finally, we analyzed the transcript levels of *StHAKs* in different tissues of potato and under various potassium fertilizer treatments.

### 4.1 Phylogenetic analysis of *StHAK* genes

The StHAKs were divided into four clades according to phylogenetic analysis (**Figure 1**), consistent with the *HAK* gene families of rice, maize and barely (Gupta et al., 2008; Zhang et al., 2012; Cai et al., 2021). The number of OsHAKs, ZmHAKs, and HvHAKs is 27 for each, with minor differences in the distribution among clades, particularly in clade II and clade III, where the number of family members is identical (Gupta et al., 2008; Zhang et al., 2012; Cai et al., 2021). The total number of PeHAKs is 41, with significantly more members in clade I and clade II compared to OsHAKs, ZmHAKs, HvHAKs, and StHAKs (Guo et al., 2024). This disparity in the number of PeHAKs raises the question of whether it is due to differences in genome sizes. For instance, the genome sizes of *mosaic bamboo*, maize, and barley are 2051 Mb, 2066 Mb, and 3800-4500 Mb, respectively (Jayakodi et al., 2020). In comparison, the genome sizes of *moso bamboo* is more than three times larger than that of the potato (DM8.1) genome (773.36 Mb), yet the number of *PeHAK* gene families is less than twice as large (Yang et al., 2023). Hence, this suggested that species genome size is not a dominant determinant for number of gene family members. The main reason, probably, is intra-genomic covariance and gene duplication events, which lead to the expansion of the *HAK* gene family members in *moso bamboo* (Guo et al., 2024). In tuber crops, the number of *StHAK* family genes (24) is slightly higher than that in *MeHAK (21)* and *IbHAK (22)* (Jin et al., 2021). Unlike the four clades of *HAK* gene family in other species, IbHAKs are divided into five clades (clade I to clade V), with clade II further subdivided into A, B and C groups. This is inconsistent with other crops like *Arabidopsis*, rice, and cassava, which have only A and B groups in clade II. Meanwhile, *OsHAK3* and *OsHAK7*, which were affiliated with clade IIA, were not classified in the phylogenetic analysis of IbHAK. This discrepancy may be due to the methods used in construction of phylogenetic tree, which showed a significance of difference between the Maximum likelihood method (ML) and Neighbor joining method (NJ) (Jin et al., 2021).

After aligning protein sequences of HAKs from different species using ClusterW, some blank regions appeared in the 5’ and 3’end of the StHAK23 and StHAK24 sequences. To ensure accuracy in our phylogenetic analysis of HAK family proteins from various species, we removed these blank regions before constructing the phylogenetic tree. The result showed that both StHAK23 and StHAK24 belonged to Clade IVB (**Figure 1**). Interestingly, AtHAK5, classified as clade I, but was neither fall into IA nor IB, and previous studies has classified it as a separate group, named IC (Gupta et al., 2008). In *cassava*, AtHAK5 is grouped into clade IB, while it is placed in clade IA in *moso bamboo* (Ou et al., 2018; Guo et al., 2024). We used ClusterW to align the amino acid sequence of AtHAK5 with OsHAKs and ZmHAKs, and the results suggested that it clusters with StHAK22, ZmHAK5, and OsHAK5, all of which belong to clade IB (**Figure 1**). Interestingly, similar to StHAK23 and StHAK24, AtHAK5 also presented blank regions at the 5’ and 3’ ends of the CDS sequences. After truncating these blank regions and constructing a phylogenetic tree, we found that AtHAK5 clustered with StHAK23 and StHAK24 in clade IVB. Although these results were inconsistent with most previous reports, the reliability and confidence of the constructed tree were still high.

### 4.2 Gene structures and conserved domains analysis of *StHAKs*

The gene structures of barley *HvHAKs* consist of 3-10 exons and 2-9 introns. While the gene structures of *PeHAKs* exhibits significant variability, ranging from 1-10 exons and 2-10 introns. The majority of *MeKUP* genes harbored 6 to 10 exons. Similarly, the gene structures of the 22 *StHAKs* comprise 6-11 exons, of which 10 genes containing 8 exons and 4 genes containing 9 exons. Distinctly, *StHAK24* contains only 1 exon, while *StHAK12* has 18 exons. The majority of *StHAKs* owned 6-10 introns, with the exceptions of *StHAK24*, which has no introns, and *StHAK14*, which has only 5 introns. The differences in exon/intron organization among *HAK* genes may provide insight into the functional diversity and evolutionary adaptation of these genes. In plants, exon/intron structure can influence gene expression regulation, mRNA splicing efficiency, and stability under various environmental conditions. Genes with more exons, like *StHAK12*, may undergo alternative splicing, potentially producing multiple protein isoforms that allow plants to flexibly respond to different stresses, including nutrient availability and abiotic stress (Laloum et al., 2018). Moreover, the introns may also play a crucial role in mediating regulation of gene expression (Rose, 2008). Tan have preformed a large-scale of bioinformatics analysis of *GT47* gene family, and identified two Clades of intron-poor genes with putative functions in drought stress responses and seed development in maize (Tan et al., 2018). These findings suggest that exon/intron organization can influence gene responsiveness under different environmental conditions, which may be particularly crucial for plant adaptation and stress responses.

To predict the conserved structural domains of StHAKs, three databases (SMART, HMMER, CDD) were chosen (**Table 1** and **Figure 2**). The results predicted by SMART and HMMER databases were similar, as all StHAK proteins contained ‘K_trans’ domain, indicating that these genes belong to the *HAK/KUP/KT* superfamily. In NCBI’ s CDD database, there are seven types of K_trans superfamily (cl15781) structural domains, including pfam02705, TIGR00794, PLN00149, PRK10745, PLN00148, PLN00150, PLN00151. Most StHAK proteins contained the ‘K_trans superfamily’ domain, and StHAK1, StHAK10, StHAK12, StHAK19 specifically contained the ‘PLN00151’ domain. In addition to the ‘PLN00151’ domain, StHAK12 also contained a domain belonging to the cl06777 superfamily, which corresponds to Rrp15p, however, detailed information about this specific protein superfamily was not available in NCBI’ s CDD database. Furthermore, similar to StHAK6, StHAK7 and StHAK11, which contained the ‘K_trans (pfam02705)’ and’PLN00149’domain respectively, the HvHAK and CqHAK proteins also possessed the ‘K_trans superfamily’, ‘PLN00151’ and ‘K_trans (pfam02705) ‘ domains (Cai et al., 2021; Chen et al., 2023b). Finally, the number of motifs predicts in different species is variable, with 10 motifs in potato, 3 in both rice and maize, 7 in quinoa, 10 in barley and mosaic bamboo, and 16 in cassava. This variation in the number of motifs may be due to differences in screening criteria (E values) or the limitations on the maximum number of motifs.

### 4.3 Analysis of collinearity, synteny, and cis-acting elements of *StHAK* genes

To further understand the evolutionary patterns of *HAK* genes in potato, the collinearity and synteny analysis were conducted on *StHAKs* both within the potato genome and between potato and other crops. We identified a total of six co-linear gene pairs of the HAK gene family within the potato genome, which located primarily on chromosomes 5, 8 and 12 (**Figure 4**). Synteny analysis between species revealed that StHAKs shared more covariant gene pairs with *GmHAKs*, *MeHAKs* and *SlHAKs*, while fewer covariant gene pairs were found with monocotyledon like *ZmHAKs*, *OsHAKs* and *SbHAKs* (**Figure 5**). These findings suggested that the genomes of potato, soybean, cassava and tomato shared many homologous gene pairs within the *HAK* gene family, indicating a closer genetic relationship among these species. In dicotyledons, *StHAK4* and *StHAK11*, located on chromosomes 2 and 5 respectively, appeared more frequently in covariant gene pairs and formed more gene pairs, whereas *StHAK13* on chromosome 6 appeared more frequently in several monocotyledons. This suggested that these homologous genes may have originated from a common ancestor and have remained relatively conserved over the span of the species evolution. The study of the synteny and evolutionary relationships of the *HAK* gene family members in potatoes not only deepens our understanding of the genetic mechanisms of potatoes but also provides precise strategies for breeding programs, including gene mapping, parental selection, and marker-assisted selection. Additionally, it lays a solid foundation for optimizing biotechnological methods such as gene editing, transgenic operations, and gene expression regulation, significantly promoting the genetic improvement of potato traits and their agricultural applications.

In this study, the elements in the promoter regions of *StHAKs* were categorized into four types based on their functions. The most numerous were light-responsive element (55.4%), which is similar to *HvHAK* (42.3%) but opposite to OsHAK and ZmHAK. Among the light-responsive elements, Box4 was the most prevalent (**Figure 6**) (Gupta et al., 2008; Zhang et al., 2012; Cai et al., 2021). The proportion of stress response elements was higher in *CqHAK* and *PeHAK* (45.3% and 14%, respectively), but lower in potato and barley (15% and 9.6%) (Cai et al., 2021; Chen et al., 2023b; Guo et al., 2024). The number of promoter/enhancer elements (CAAT box and TATA box) in *StHAKs* was not analyzed, whereas it was 13.5% and 52% in *HvHAKs* and *PeHAKs*, respectively (Cai et al., 2021; Guo et al., 2024). Interestingly, the Ca^2+^ response elements was numerically and widely distributed in the promoter regions of *OsHAKs* and *ZmHAKs*, but were absent in the promoter regions of *StHAK* and *HvHAK* (Gupta et al., 2008; Zhang et al., 2012; Cai et al., 2021). In the promoter regions of *StHAKs*, the most relevant cis-acting elements to stress response are ARE (Anaerobic Responsive Element), MBS (MYB Binding Site) and LTR (Low-Temperature Responsive Element), and the most relevant cis-acting elements to hormone regulation are ABRE (Abscisic Acid Responsive Element), TGA-element (Auxin Responsive Element), TATC-box (Cytokinin Responsive Element), P-box (Gibberellin Responsive Element), CGTCA-motif (Methyl Jasmonate Responsive Element), TGACG-motif (Methyl Jasmonate Responsive Element), TCA-element (Salicylic Acid Responsive Element). As reported, that MYB77 directly bind to MYB factor binding motifs MYBcore (CNGTTR) and MRE(AACC), which located in the *HAK5* gene promoter region to involve in potassium uptake (Feng et al., 2021). Four transcription factors dwarf and delayed flowering 2 (DDF2), jagged lateral organs (JLO), basic helix-loop-helix 121 (bHLH121) and transcription initiation factor II_A gamma chain (TFII_A) induced the expression of *HAK5* via binding to the HAK5 promoter when plants were grown under low K conditions, while the specific cis-acting elements they bind to were still not clearly defined (Hong et al., 2013; Mulet et al., 2023). Although various transcription factors (TFs) that respond to abiotic stress signals have been reported to induce the expression of *HAK* genes, specific motifs within the *HAK* gene promoter regions targeted by these TFs remain largely unidentified. In terms of hormone signaling, low potassium levels also trigger responses involving phytohormones such as cytokinin, auxin, jasmonic acid (JA), and ethylene (ET) (Johnson et al., 2022). *HAK5* expression, for instance, is upregulated by reactive oxygen species (ROS) and ethylene signals under potassium-deficient conditions (Schachtman, 2015). Another K^+^ transporter, OsCHX14, which is preferentially expressed in flowers, is regulated by JA (Chen et al., 2016). Although similar findings exist, these studies have yet to clarify the specific upstream and downstream regulatory interactions, especially regarding the functional role of specific motifs within the HAK promoter region, such as the CGTCA-motif, TGACG-motif, and P-box. Understanding how these motifs are involved in hormone or stress-induced regulation of HAK gene expression remains limited (Hong et al., 2013; Armengaud et al., 2004; Johnson et al., 2022). Therefore, research focused on hormone- or stress-induced motifs that regulate HAK gene expression could provide significant insights.

### 4.4 Expression of *StHAK* genes across various tissues and their responses to different stresses

A well-understanding of the expression properties of *StHAKs* is crucial for elucidating their functions. In the study, the transcript levels of *StHAKs* in diverse tissues of potato and under different potassium fertilizer treatments were assayed through publicly available RNA-Seq data, transcriptome data and qRT-PCR. Microarray data revealed that most *StHAKs* in clade I and IV were expressed at relatively low levels across all organs, while those in clade II and III showed higher expression levels in all test tissues (**Figure 7**). These results are consistent with studies on other species such as *Arabidopsis*, barley, and quinoa (MaüSer et al., 2001; Cai et al., 2021; Chen et al., 2023b). Notably, *StHAK18* and *StHAK20* were exclusively expressed in carpels, stamens and mature flowers of potato, indicating a specific role in regulating the reproductive growth. The high mRNA levels of *StHAK8* in stolons, compared to other tissues, indicated its potential involvement in potato tuber formation. Transcriptomic data revealed that *StHAK4*, *StHAK9*, and *StHAK24* were more abundant in leaves and roots than in tubers, suggesting their involvement in K^+^ uptake by roots, photosynthesis in leaves, and photosynthate allocation in potatoes. This requires further experimental validation. Chen et al. (2017) showed that *OsHAK1* is highly expressed in rice root tips but weakly in aerial parts, indicating its key role in root K^+^ uptake. According to qRT-PCR results, *StHAK14* was expressed significantly in tubers, suggesting that it may be involved in the development of potato tubers (**Figure 10B**). This finding was somewhat not the same with RNA-seq or transcriptome data, which did not show a apparent expression of *StHAK14*. *StHAK14* shared 48% similarity with *AtKUP8*, which maintains K^+^ homeostasis and responds to heavy metal stress (e.g., Cd, Cr and Cu) in *Arabidopsis* (Sanz-Fernandez et al., 2021). *AtKUP8* is also involving in promoting cellular osmotic adaptation by maintaining K^+^ homeostasis under drought conditions, while the exact function of *StHAK14* in tubers remains unclear, which needs to further investigate (Osakabe et al., 2013). *StHAK4* was abundant in potato leaves, consistent with both qRT-PCR and transcriptomic data (**Figures 8**, **10**). *StHAK8* was enriched in stolons in RNA-seq, and also expressed obviously in stems and tubers according to qRT-PCR results (**Figures 7**, **10**). Additionally, qRT-PCR results showed that *StHAK18* and *StHAK21* were apparently expressed in flowers, which consistent with Microarray findings (**Figures 7**, **10**).

Different paralogs of *StHAKs* show significant expression in various tissues such as roots, stems, leaves, stolon, tubers and flowers (including carpels, stamens, sepals, petals), as indicated in the results. As previously reported, *AtHAK5* is chiefly expressed in roots and regulates K^+^ uptake under low potassium conditions, while *AtHAK4* and *AtHAK9* are primarily expressed in stems and leaves, suggesting their involvement in K^+^ transport and distribution (Ahn et al., 2004). Similarly, in rice, genes such as *OsHAK1* and *OsHAK2* are expressed in roots to promote K^+^ absorption, while *OsHAK5* and *OsHAK16* are expressed in leaves, likely contributing to K^+^ redistribution and leaf function. *OsHAK10* and *OsHAK15* are expressed specifically in stamens, suggesting they play key roles in pollination or fertilization (Gupta et al., 2008). In cassava, five genes including *MeKUP3* and *MeKUP4*, are broadly expressed in stems, leaves and store roots, while *MeKUP1*, *MeKUP19*, and *MeKUP20* are expressed significantly only in leaves, indicating their involvement in K^+^ redistribution (Ou et al., 2018). This suggest that: (1) The *HAKs* gene family is widely involved in critical physiological processes, such as K^+^ absorption and distribution, and the development of vegetative organs (e.g., tubers) and reproductive organs (e.g., flowers); (2) Different *HAKs* paralogs perform specific biological roles in distinct tissues, optimizing K^+^ uptake and utilization under diverse environmental conditions. Therefore, investigating the expression profiles of *HAKs* paralogs can provide insights into how these genes regulate K^+^ homeostasis through gene differentiation and contribute to plant adaptation to environmental changes. This work also provides valuable information for a deeper understanding of *HAKs* gene functions.

Moreover, *StHAKs* are crucial in responding to abiotic stresses. As we can see, *StHAK1, StHAK6, StHAK11*, and *StHAK22* responded to at least two abiotic stresses, while *StAHK14* was able to respond to three (salt, mannitol, and heat) (**Figure 7B**). As reported previously, the expression levels of *AtHAK2*, *AtHAK6* and *AtHAK11* were significantly raised under high salt stress (Maathuis, 2006). Likewise, the relative abundance levels of three and eight *TaHAKs* are dramatically up-regulated under salt and drought stress, respectively (Cheng et al., 2018). Most *HAK* gene family members in *moso bamboo* are capable of responding to at least two abiotic stresses (Guo et al., 2024). In addition to salt and mannitol stress, *StHAK6* was also induced by IAA (**Figure 7B**). *StHAK20* and *StHAK21* were co-induced by IAA and GA3, however, *StHAK20* was suppressed by ABA (**Figure 7B**). Like *StHAK20*, some *PeHAKs* are involved in the cross-pathways of multiple hormone signals, such as ABA and IAA, or NAA and GA (Guo et al., 2024). Moreover, certain *StHAKs*, such as *StHAK4* and *StHAK9*, were also somewhat capable of responding to biotic stresses (**Figure 7B**).

K^+^ is a crucial ion in regulating stomatal movement in plant (Zhang et al., 2020). Stomatal closure is triggered by the efflux of K^+^ from guard cells, leading to water loss from the cells, which causes them to deflate and closes the stomata. This process is critical during drought conditions to reduce water loss through transpiration. As we all known, potassium transporters facilitate this movement of K^+^. *HAKs* (High-affinity potassium transporters) play a significant role in potassium uptake, particularly under low potassium conditions, which can be exacerbated during drought stress. Studies on *Arabidopsis* and wheat have shown that specific members of the HAK family are involved in transporting K^+^ from roots to shoots, ensuring that adequate K^+^ levels are maintained in guard cells to modulate stomatal movements under stress. The similar mechanisms may be expected in potato, although specific research on *StHAK* members like *StHAK5* remains limited. Potatoes, like many plants, rely on K^+^ for maintaining turgor pressure in guard cells to regulate stomatal aperture. Under drought conditions, potassium transporters such as HAKs and KUPs likely play a critical role in maintaining this function, thus reducing water loss. Further experimental evidence, such as gene expression studies under drought conditions, could provide more specific insights into which K^+^ transporters are most active during drought stress in potato. The future studies would be focused on elucidating the specific roles of potato HAK/KUP transporters in stomatal regulation during drought stress. Some experiments such as gene expression analysis, knockout studies, and physiological assays under drought conditions need to conduct, which could provide clearer insights into these transporters’ functional roles. In summary, potassium transporters are integral to stomatal regulation, and evidence from other species suggests that potato HAK transporters may perform similar functions. Of course, this hypothesis requires further validation.

Based on our in-depth analysis of the *HAK* gene family in potatoes, we believe these genes have significant potential in regulating potassium ion balance and enhancing drought and cold resistance, as well as tolerance to high light intensity in crops. In the future, gene-editing techniques (like CRISPR/Cas9) could be used to optimize the expression of these key genes, thus enabling the development of potato or other crops with improved stress resilience. Additionally, these findings could serve as targets for improving stress tolerance in other crops, providing new approaches to address global climate change challenges and enhance the stability of agricultural production.

## 5 Conclusion

In this study, a total of 24 *StHAK* genes were identified and divided into four subfamilies with potentially similar functions. The expression levels of these *StHAKs* varied significantly across different tissues and displayed a obvious difference under various abiotic stress, hormone treatments and potassium fertilizer conditions. This suggests that these genes were expressed tissue-specificity as well as are involved in both abiotic stress response and potassium uptake. To clearly display *HAK* gene expression across potato tissues, we created a cartoon heatmap illustrating their tissue-specific patterns, an unprecedented approach in potato gene family analysis. Finally, *StHAK* genes like *StHAK8*, *StHAK14*, and *StHAK22* as highly expressed in tubers, suggesting their role in tuber growth and development. Consequently, these findings would contribute to a deeper understanding of the biological functions of *HAK/KUP/KT* genes, and provide a solid basis for further functional analysis of these genes in potato growth and potassium responses.

## Data Availability

The original contributions presented in the study are included in the article/**Supplementary Material**, further inquiries can be directed to the corresponding author/s.
