# Supplementary figures and images for: Genome-wide identification, characterization and expression pattern analysis of HAK/KUP/KT potassium transporter gene family in potato
Source: Front Plant Sci. 2025 Jan 16;15:1487794. doi: 10.3389/fpls.2024.1487794 (PMC11779732; doi:10.3389/fpls.2024.1487794)

Tree scale: 1

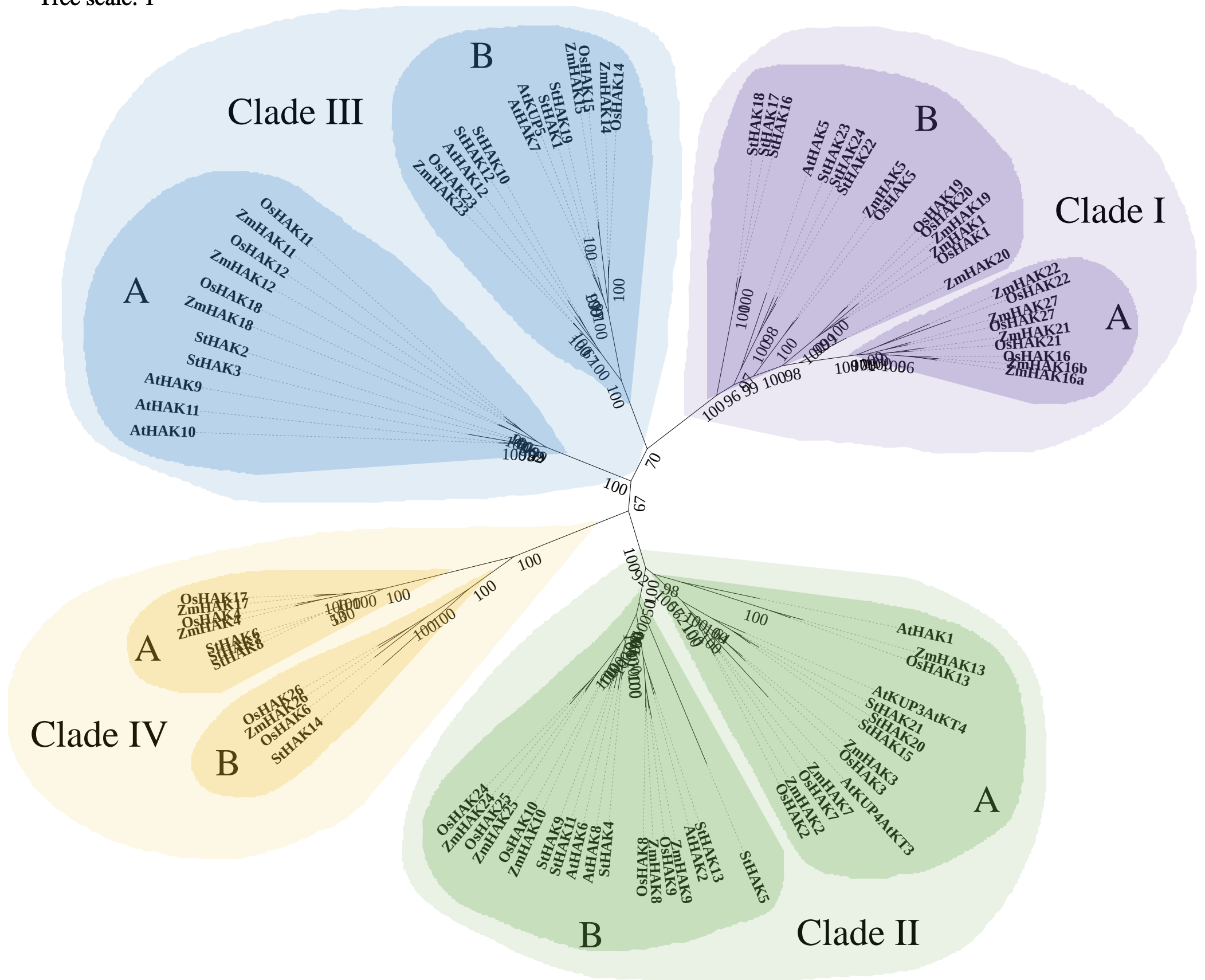

Supplement: Supplementary file 1 [file DataSheet1.pdf]
