# Supplementary material for: Genome-wide identification, characterization and expression pattern analysis of HAK/KUP/KT potassium transporter gene family in potato
Source: Front Plant Sci. 2025 Jan 16;15:1487794. doi: 10.3389/fpls.2024.1487794 (PMC11779732; doi:10.3389/fpls.2024.1487794)
Supplement: Supplementary file 4 [file Table3.docx]

Supplementary Table 3: Conserved motifs identified from the *StHAK* genes in potato*.*

| Motif1 | \| 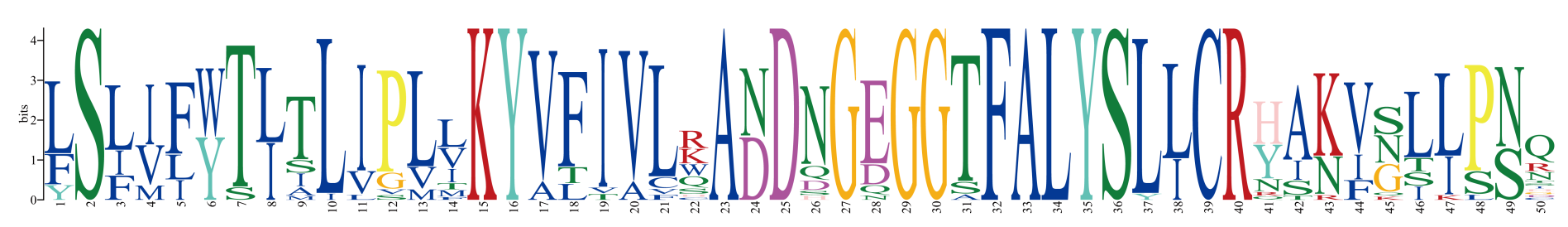 \| \| --- \| |
| --- | --- | --- |
|  | LSLIFWTJTLIPLLKYVFIVLRABDNGEGGTFALYSLLCRHAKVNLLPNQ |
| Motif2 | 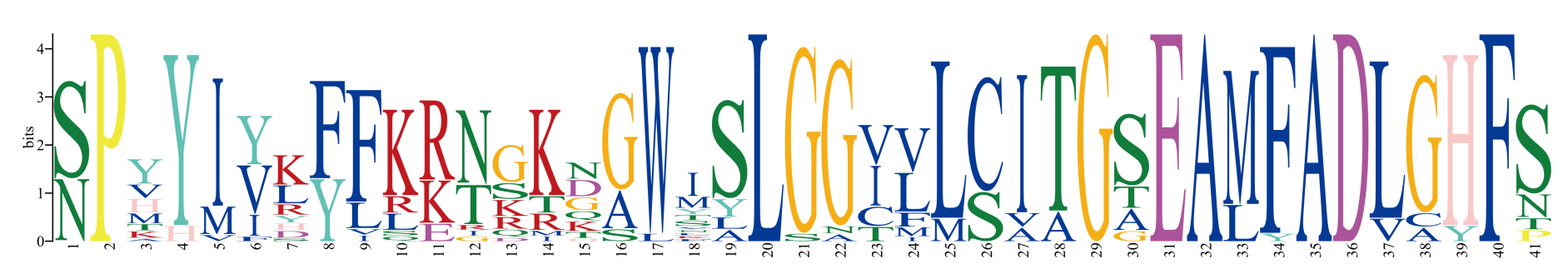 |
|  | SPYYIVKFFKRNGKNGWISLGGVVLCITGSEAMFADLGHFS |
| Motif3 | 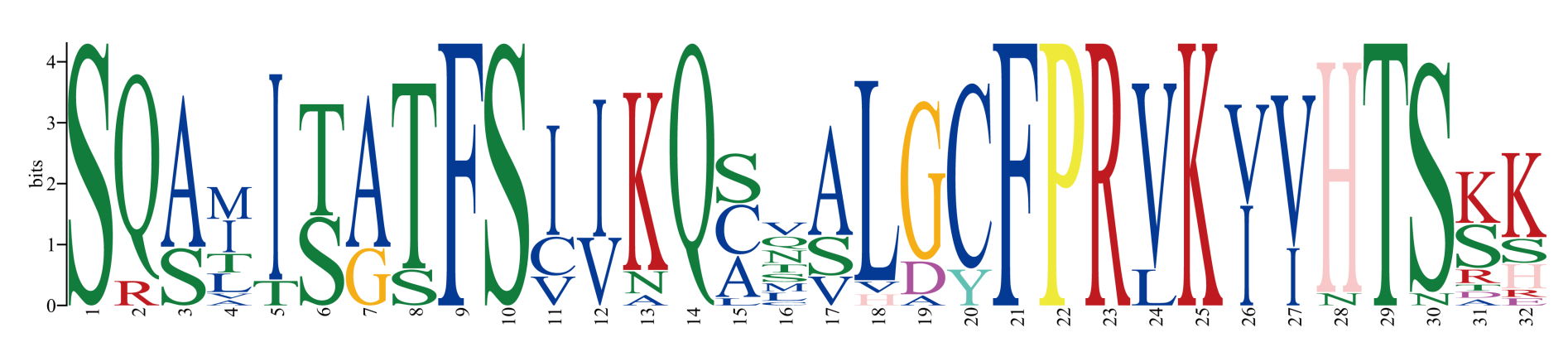 |
|  | SQAIITATFSIIKQSVALGCFPRVKVVHTSKK |
| Motif4 | 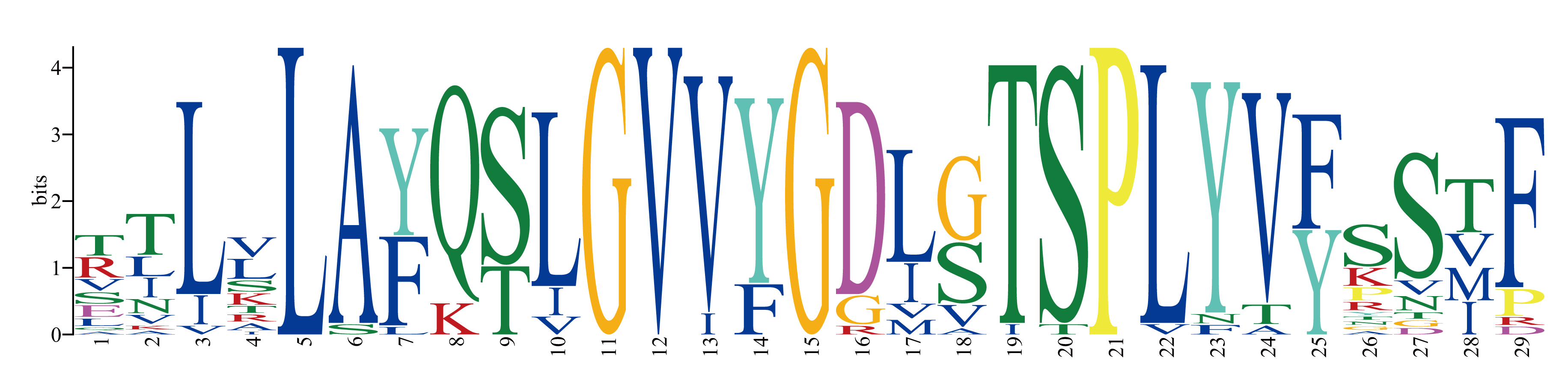 |
|  | TTLVLAYQSLGVVYGDLGTSPLYVFSSTF |
| Motif5 | 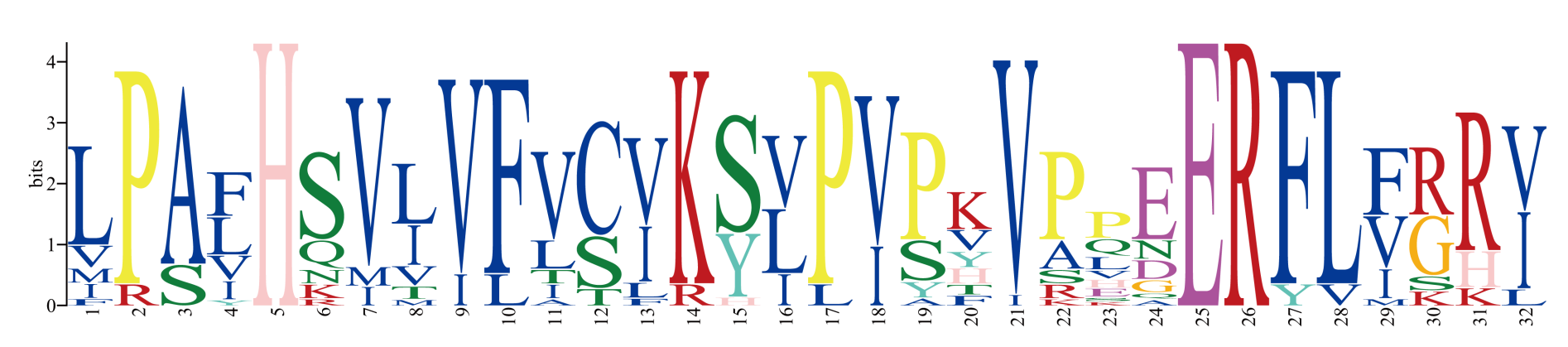 |
|  | LPAFHSVJVFVCVKSVPVPKVPPEERFLFRRV |
| Motif6 | 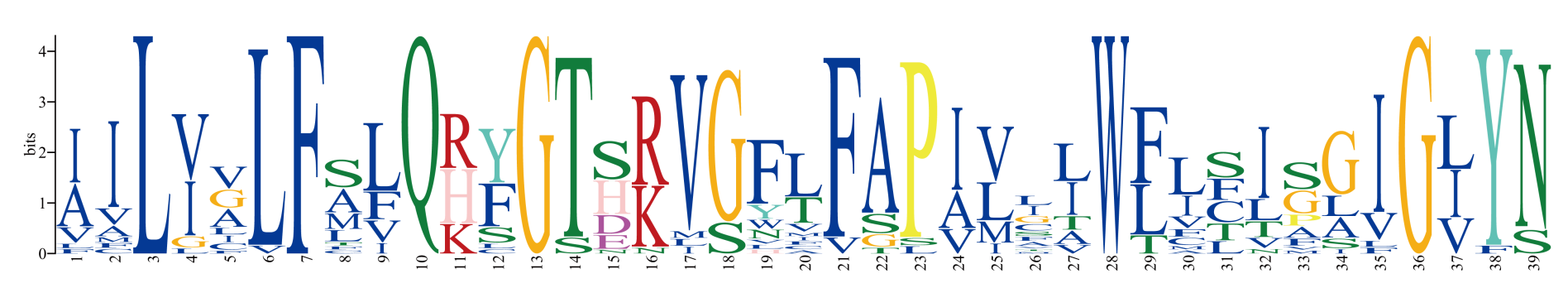 |
|  | IILVVLFSLQRYGTSRVGFLFAPIVLJWFJSISGIGLYN |
| Motif7 | 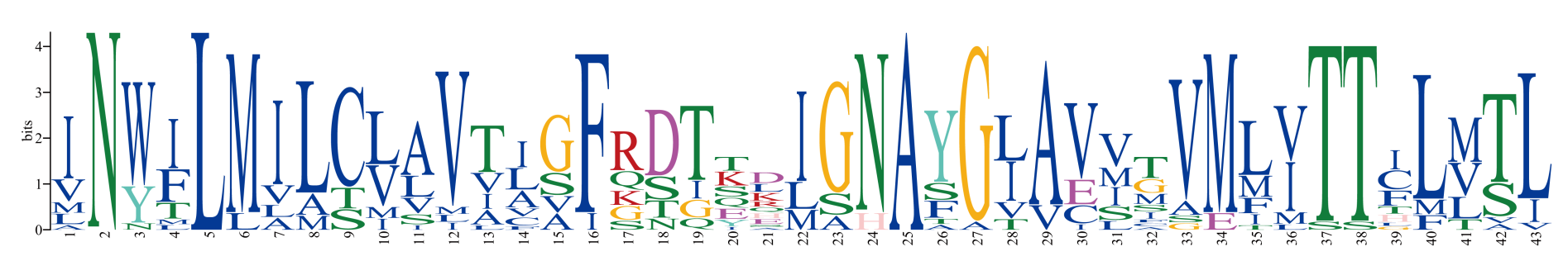 |
|  | INWILMILCLAVTIGFRDTKDIGNAYGJAVVTVMLVTTILMTL |
| Motif8 | 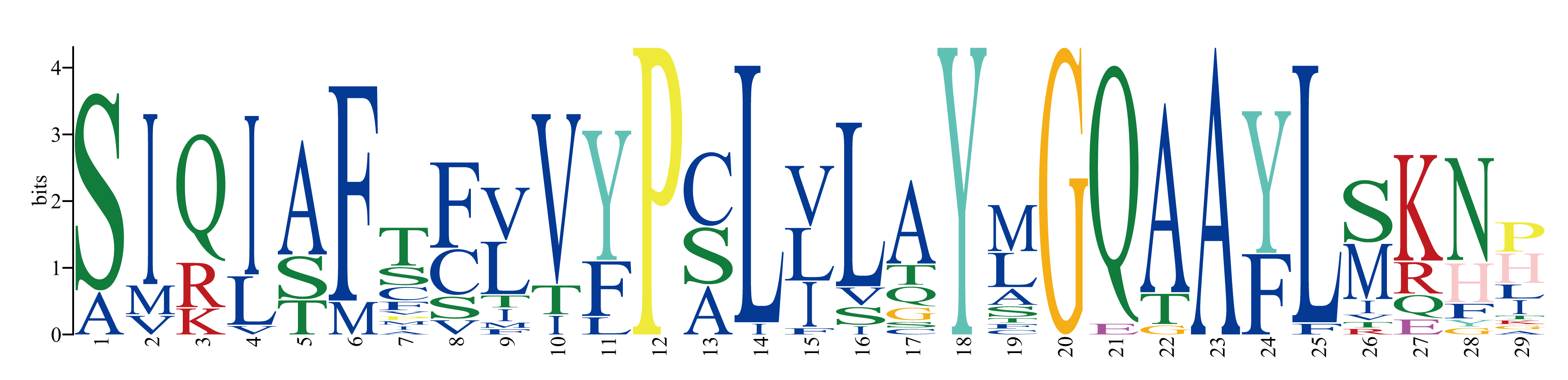 |
|  | SIQIAFTFLVYPCLVLAYMGQAAYLSKNP |
| Motif9 | 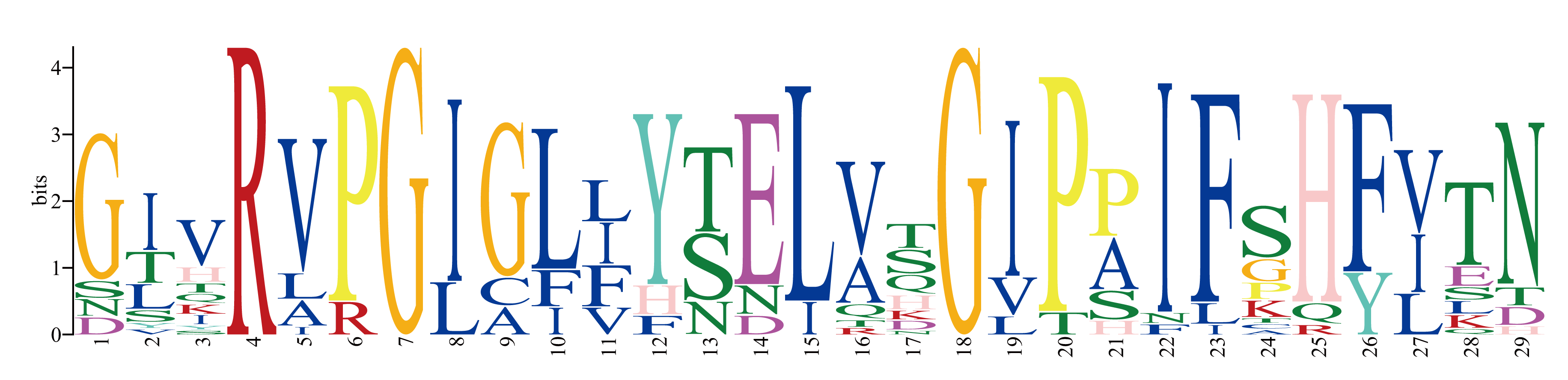 |
|  | GIVRVPGIGLLYTELVSGIPPIFSHFVTN |
| Motif10 | 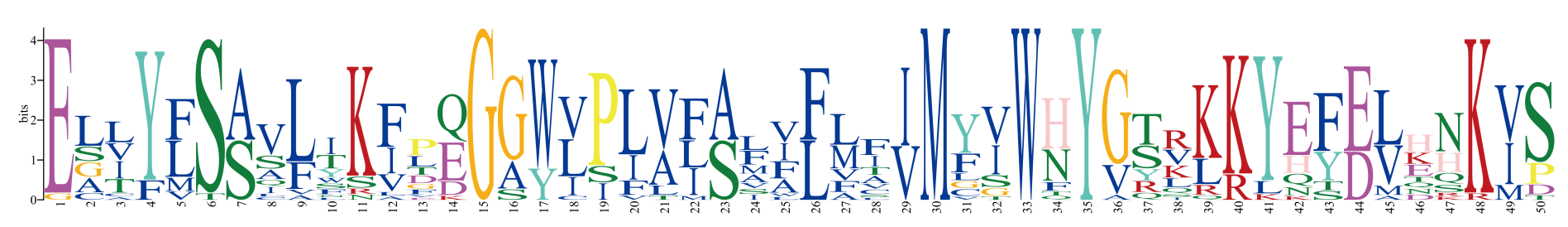 |
|  | ELLYFSAVLIKFPZGGWVPLVFALVFLFIMYVWHYGTRKKYEFELHNKVS |
